# Glyceric Prodrug of Ursodeoxycholic Acid (UDCA): Novozym 435-Catalyzed Synthesis of UDCA-Monoglyceride

**DOI:** 10.3390/molecules26195966

**Published:** 2021-10-01

**Authors:** Federico Zappaterra, Stefania Costa, Daniela Summa, Bruno Semeraro, Virginia Cristofori, Claudio Trapella, Elena Tamburini

**Affiliations:** 1 Department of Life Sciences and Biotechnology, University of Ferrara, Via L. Borsari, 46, 44121 Ferrara, Italy; zppfrc@unife.it (F.Z.); smmdnl@unife.it (D.S.); tme@unife.it (E.T.); 2 GATE SRL, Via L. Borsari, 46, 44121 Ferrara, Italy; bsemeraro@gategreen.it; 3 Department of Chemistry and Pharmaceutical Sciences, University of Ferrara, Via Fossato di Mortara, 17, 44121 Ferrara, Italy; virginia.cristofori@unife.it (V.C.); trpcld@unife.it (C.T.)

**Keywords:** ursodeoxycholic acid, monoglyceride, esterification, CALB, prodrug

## Abstract

Bile acids (BAs) are a family of steroids synthesized from cholesterol in the liver. Among bile acids, ursodeoxycholic acid (UDCA) is the drug of choice for treating primary biliary cirrhosis and dissolving cholesterol gallstones. The clinical effectiveness of UDCA includes its choleretic activity, the capability to inhibit hydrophobic bile acid absorption by the intestine under cholestatic conditions, reducing cholangiocyte injury, stimulation of impaired biliary output, and inhibition of hepatocyte apoptosis. Despite its clinical effectiveness, UDCA is poorly soluble in the gastro-duodeno-jejunal contents, and pharmacological doses of UDCA are not readily soluble in the stomach and intestine, resulting in incomplete absorption. Indeed, the solubility of 20 mg/L greatly limits the bioavailability of UDCA. Since the bioavailability of drug products plays a critical role in the design of oral administration dosages, we investigated the enzymatic esterification of UDCA as a strategy of hydrophilization. Therefore, we decided to enzymatically synthesize a glyceric ester of UDCA bile acid to produce a more water-soluble molecule. The esterification reactions between UDCA and glycerol were performed with an immobilized lipase B from *Candida antarctica* (Novozym 435) in solvent-free and solvent-assisted systems. The characterization of the UDCA-monoglyceride, enzymatically synthesized, has been performed by ^1^H-NMR, ^13^C-NMR, COSY, HSQC, HMBC, IR, and MS spectroscopy.

## 1. Introduction

Bile acids, also called C24 bile acids, belong to the family of biological steroid derivatives and have been isolated and characterized from bile since the 1950s. These acids are synthesized from cholesterol, secreted in bile, and stored in the gallbladder. Among these, an important pharmacological role is constituted by ursodeoxycholic acid (UDCA) [1]. Indeed, ursodeoxycholic acid (3α, 7β-dihydroxy-5β-cholanoic acid), also known as ursodiol, is an active ingredient widely used in clinics to dissolve cholesterol gallstones and to treat cholestatic forms of liver disease including primary biliary cirrhosis. This secondary bile acid derives from the metabolism of cholic acid by the human intestinal microbiota. Ursodeoxycholic acid regulates cholesterol levels by slowing the rate at which the intestine can absorb cholesterol and also acts to break down the micelles, which contain cholesterol [2,3]. Ursodeoxycholic acid reduces elevated levels of liver enzymes by facilitating the flow of bile through the liver and protecting liver cells [4]. UDCA has been orally administrated for the treatment of chronic liver diseases in patients with cystic fibrosis (CF)-related cholestasis [5], cholestasis correlated with pregnancy [6], sclerosing cholangitis, chronic active hepatitis, and viral hepatitis [7]. Moreover, UDCA has been tried as a therapeutic active ingredient for non-alcoholic steatohepatitis and refractory graft-versus-host disease of the liver in transplant patients [8]. Furthermore, UDCA is employed as an anti-inflammatory and antioxidant [9,10], and many UDCA derivatives have been synthesized as hybrid functional compounds with anticancer activity [11].

However, although UDCA is more soluble in water (hydrophilic) and less intrinsically toxic to cells than the primary cholic acid when orally administrated, its bioavailability suffers from the poor solubility of the molecule. Indeed, with its water solubility of only 20 mg/L, UDCA is one of the poorly soluble in water and poorly bioavailable drugs [12]. The poor solubility of UDCA creates challenges in reaching therapeutic drug concentration [13]. Due to the high pharmacological interest in this active ingredient, the literature reports various approaches aimed at improving the bioavailability of UDCA by virtue of its poor solubility.

Many efforts are being made to improve the solubility, and therefore bioavailability, of UDCA. There are formulations with UDCA salt conjugates (e.g., sodium) to enhance dissolution rates in the stomach. Poorly soluble molecules can be enhanced in water solubility using saline forms, for example, UDCA sodium salt has a water solubility of 200 g L^−1^. Several strategies are based on the design of the drug in nano- or micro-scale particles [14], spray drying [15], nano-emulsification [16], or through electrochemically controlled drug delivery systems based on tauroursodeoxycholic acid and PEDOT [17]. Furthermore, Chung et al. investigated the apparent solubility of UDCA concerning its surface polarity [18]. Thus, to improve its bioavailability, approaches to the design of UDCA prodrugs have yielded encouraging results. Dosa et al. synthesized five phosphate prodrugs of UDCA that showed significant anti-apoptotic activity in vitro [19]. Furthermore, many UDCA prodrugs exploit the use of an ester bond. A 5′-ester conjugation of azidothymidine with UDCA has been reported as an antiviral agent [20]. UDCA, conjugated to dihydroartemisinin (DHA) via ester bound has been evaluated as an anticancer in hepatocellular carcinoma (HCC) [21]. A polymeric prodrug of UDCA made by peroxalate ester linkage has antioxidant and anti-inflammatory activities and plays valuable roles in bone regeneration [22]. UDCA’s galactosylated prodrug was evaluated in terms of pharmacological effects in cholestatic rat models and it showed higher potency compared to UDCA in reducing serum biomarkers and cytokines [23]. Recently, nasal administration of nanoencapsutated geraniol/ursodeoxycholic acid conjugate has been proposed as an approach for the management of Parkinson’s disease [24].

Thus, esterification strategies can be exploited to increase the polarity and thereby improve the aqueous solubility of the lipophilic compounds, which will be covalently attached to water-soluble molecules, resulting in an ester [25]. Moreover, it has been reported that esters display good thermodynamic stability in vivo [26].

Although the reported strategies are promising, the design of these prodrugs has always envisaged the exploitation of a chemical synthesis route. Unlike the enzymatic route, the chemical route consists of a catalytic approach with a greater environmental impact. In sustainable catalysis, UDCA prodrugs can be produced by synthesizing ester bonds by exploiting biotransformations with free or immobilized enzymes. However, few literature sources report UDCA esterifications by the enzymatic route. Sugai et al. reported a lipase-catalyzed preparation of fatty acid esters of bile acids and tested their anti-bacterial activity. Nevertheless, the preparations thus employed were not intended to improve the solubility of UDCA.

The literature that sees the use of lipases as serine proteases biocatalyzing the ester bound is vast. Lipase-catalyzed esterification reaction has called attention since the 1990s. Lipase (triacylglycerol hydrolases, EC 3.1.1.3) for the esterification reaction was employed due to their noteworthy activity, selectivity, and mild operating conditions. Due to the tolerance towards organic solvents, lipases were applied in fine chemical industries, specifically in the presence of thermosensitive products [27].

In recent times, many efforts have been employed in the use of these biological catalysts in difficult-to-use contexts, such as, for example, those that see the union between poorly water-soluble molecules and highly hydrophilic molecules. The coexistence of two chemical species with such different polarities in a single reaction environment is difficult. Despite this, it has been reported that polyalcohol can be exploited as hydrophilization moieties for the synthesis of esters with increased polarity and therefore solubility. Ibuprofen, a non-steroidal anti-inflammatory drug NSAID with poor solubility in water, was effectively esterified with the polyalcohol sorbitol through a reaction biocatalyzed by the free porcine pancreas lipase enzyme [28]. The same polyalcohol has also been used in the hydrophilization of bixin [25]. Although the hydrophilizing capacity of sorbitol polyalcohol is good, its solid nature limits the use and conversion yield of the process. In fact, it is necessary to use biphasic systems that involve the use of water for the solubilization of ibuprofen, and this is a limiting factor for the conversion of the substrate, leading the lipase to produce hydrolysis of the newly formed bond. To overcome some of these limitations, useful biocatalytic strategies, called solventless, have allowed the covalent attach of poorly bioavailable active ingredients with the polyol glycerol to produce prodrugs with increased solubility and bioavailability. In fact, since glycerol is a highly viscous polyalcohol in liquid form, it takes part in the esterification reaction both as a reagent and as a solvent.

Glycerol, propane-1,2,3-triol, is a short polyalcohol with three hydroxylic groups. This nontoxic viscous liquid is highly hygroscopic, has a high boiling point, and low vapor pressure. It is extensively used in the cosmetic, chemical, pharmaceutical, and food industries [26]. Due to the great increase in biodiesel production, glycerol availability grew enormously in recent years, as reported by the international environmental laws (IEL). Indeed, biodiesel manufacturing processes involve glycerol as the main processing by-product, being about 10% *w*/*w* of biodiesel. Thus, by virtue of its availability and low cost, the uses of glycerol have multiplied, extending beyond uses in food, cosmetic, and pharmaceutical fields. One of these, for example, involves the use of glycerol as a useful raw material for designing protocols that exploit its ability to act both as a solvent and as a reagent in lipase-catalyzed enzymatic syntheses [29]. Among the advantages of glycerol in solventless systems, there is the well-recognized phenomenon of this, as of other polyalcohols, of conferring stability to the enzymatic catalyst [30]. Indeed, polyalcohols, such as glycerol, are often used as cosolvent for protein stabilization [31]. The native protein structure of the catalyst can be shifted by glycerol to a more compact state [32]. This interesting phenomenon can prevent the loss of enzymatic activity, expand the thermal unfolding temperature, and avoid irreversible aggregation of proteins, making the enzymatic synthesis protocols that involve the use of this short polyalcohol particularly flexible [33]. Furthermore, solventless systems with glycerol allow emancipation from organic solvents by exploiting this polyalcohol as non-toxic, biodegradable, and recyclable green solvent for high product yields and selectivity in catalysis and enzymatic catalysis [34,35].

Several substrates belonging to different industrial sectors have been enzymatically esterified to form mostly monoglycerides. Ravelo et al. [36] and Tamayo et al. [27] reported the monoglyceride synthesis of ibuprofen and benzoic acid, respectively. Zappaterra et al. reported the synthesis of glycerol sorbate by covalent to attach sorbic acid with glycerol as a potential strategy for antimicrobial control in agri-food industries [37]. Therefore, the esterification system with lipase-catalyzed glycerol has been proven to be an exploitable strategy, both in pharmaceutical and food preservation contexts, for the enhancement of poorly bioavailable active ingredients. In these protocols, the well-known biocatalyst *Candida antarctica* lipase type B (CALB) was tested.

In most cases, in the enzymatic reactions in a monophasic environment, it is necessary to use this catalyst. Indeed, several works in scientific literature report the use of *C. antarctica* lipase B efficiently performing esterification reactions in monophasic [38]. The reason why CALB is the most exploited enzyme for solventless enzymatic esterification reactions resides in its tiny lid which closes only partially the active site of the enzyme [39], making interfacial activation unneeded. For these reasons, CALB has formerly proved to be the right choice to work systems lacking the solvent–water interface [27], i.e., monophasic systems [40]. Both free and immobilized forms of this enzyme are available for this enzyme [41]. The immobilized form of this enzyme stood out for its stability and catalytic activity in a wide range of organic solvents [42]. This peculiar stability of CALB in an immobilized form, which makes it the most stable enzyme on the market, facilitates its use for industrial applications [43].

Hence, we aimed to design solventless- and solvent-assisted media strategies to perform the lipase-catalyzed esterification of UDCA with glycerol to obtain an UDCA prodrug with greater polarity than the starting active ingredient. In these reactions, glycerol acts both as a reagent and as a solvent.

## 2. Results and Discussion

UDCA, with its water solubility of only 20 mg L^−1^, is one of the poorly-soluble active ingredients and, therefore, poorly bioavailable [44]. The union of this active ingredient with a hydrophilizing function can lead to the development of a prodrug with a potential increased water-solubility.

In this work, the esterification of UDCA with glycerol is presented to produce the UDCA-monoglyceride prodrug. For this purpose, an enzymatic catalyst, immobilized type B lipase from *C. antarctica* (N435), was chosen.

Ursodeoxycholic acid has a catalytically available carboxylic acid group that can be targeted for the direct esterification reaction catalyzed by N435, resulting in a covalent attachment of the glycerol hydrophilization moiety. One of the advantages of choosing the enzymatic synthesis pathway compared to which involves the use of chemical catalysts lies in the enzymatic regiospecificity that allows for the avoidance of the protective steps of the reactive groups. Moreover, the enzymatic catalysis allows for operating in mild reaction conditions.

**Scheme 1:**
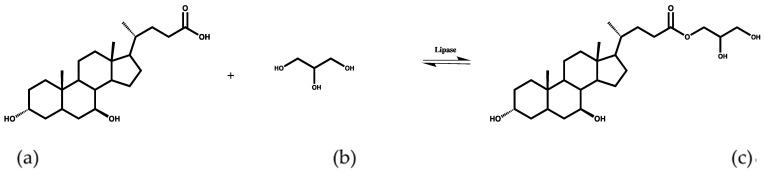
The reaction between ursodeoxycholic acid (UDCA) and glycerol catalyzed by immobilized lipase B from *C. antarctica*: (**a**) UDCA, (**b**) glycerol, (**c**) glycerol ester of UDCA.

As reported in Scheme 1, the covalent attachment of glycerol to UDCA creates a new stereogenic center at the C2 position of glycerol. Canonically, in esterification that involves a biocatalyst (in this specific case a lipase), the enzyme is not able to discriminate between the two primary hydroxyls of the polyol (in this case glycerol), which will be equivalent. Thus, the enzymatic synthesis of UDCA-monoglyceride, catalyzed by N435, produced the mixture of the two epimers. Due to the chiral center of glycerol in C2, the mixture of epimers will be partitioned with an R/S ratio of 50:50. For this reason, we chose not to report chirality in Scheme 1.

For effective production of the esterification of UDCA with glycerol, fine management of the reaction environment is required. Indeed, the liquid-viscous nature of glycerol allows for the development of both solventless and solvent-assisted systems, both biphasic and monophasic.

To the best of our knowledge, although there are prodrugs of UDCA, no sources in the literature report the synthesis of the UDCA-monoglyceride prodrug, neither through enzymatic nor chemical catalysis.

For the enzymatic production of UDCA-monoglyceride, we studied several esterification parameters to reach the operating conditions: solubility of UDCA in solventless and *solvent-assisted* medium, enzyme stability, enzyme concentration, temperature, stirring speed, and reaction time. To evaluate the effects of changes in the parameter, we studied the conversion yield of the substrates. The purified product was analyzed using ^1^H-NMR, ^13^C-NMR, COSY, HSQC, HMBC, IR, and uHPLC–MS.

### 2.1. Preliminary Experiments: Choice of the Suitable Organic Co-Solvent

The abundance and consequent low price of glycerol make it a perfect green solvent that, in our protocol, acts both as a solvent and as a reagent in the UDCA esterification reaction. The literature reports how this small polyalcohol can be used for the solubilization of poorly water-soluble substrates such as ibuprofen [26] and sorbic acid [37] in systems known as solventless. Furthermore, it is possible to employ strategies that involve the use of an organic solvent that, in the medium, acts as a co-solvent by increasing the solubilization of the active ingredient of interest and decreasing the viscosity of glycerol [36]. Depending on whether the co-solvent is miscible with glycerol, both biphasic and monophasic solvent-assisted (SA) systems can be developed.

For this purpose, we evaluated which of the three strategies to employ (solventless, solvent-assist biphasic, or monophasic), by studying two high-boiling organic solvents, the immiscible with glycerol, toluene, and the miscible 2-methylbutan-2-ol. The characteristics that led us to use these co-solvents are shown in Table 1.

Among the suitable organic solvents for enzymatic esterification, we focused on the two high-boiling solvents: toluene and 2-methylbutan-2-ol. These solvents, due to their high boiling point, would have allowed us to operate in a wide range of temperatures, representing a net experimental benefit. In fact, N435 is a very flexible catalyst capable of operating at high temperatures. These organic solvents, with a boiling point above 100 °C, do not undergo evaporation at the working temperatures of the enzyme, making them suitable for a batch process.

To evaluate whether to exploit a biphasic glycerol/toluene strategy or a monophasic strategy with 2-methylbutan-2-ol as co-solvent, we evaluated the ability of these organic solvents to solubilize UDCA. At 65 °C, 2-methylbutan-2-ol was found to be capable of solubilizing UDCA 10 times more than toluene. The solubility of UDCA in 2-methylbutan-2-ol was over 100 g L^−1^. Thus, we decided to continue with monophasic strategies and evaluate the solubility of UDCA in glycerol with and without the use of the co-solvent 2-methylbutan-2-ol.

### 2.2. Solubility of UDCA: Solventless and Solvent-Assisted Media

**Figure 1:**
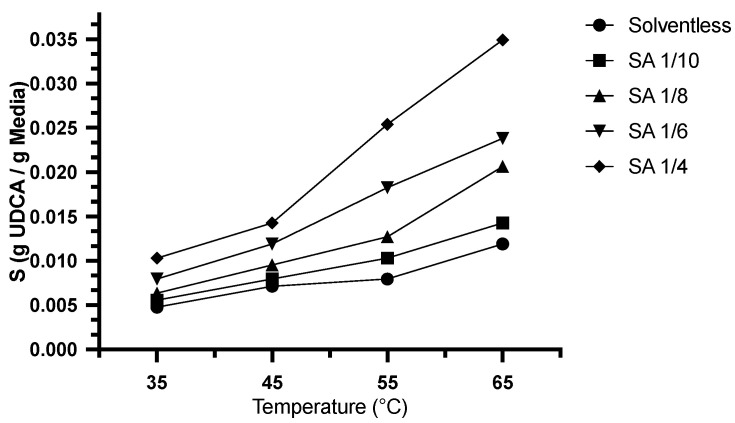
Solubility of UDCA in solventless and solvent-assisted conditions.

The solubility of the acid substrate in the reaction medium of enzymatic biotransformation is a decisive parameter for the success of the esterification process, especially when using an immobilized enzyme catalyst. To evaluate whether to use a solventless system or to use 2-methylbutan-2-ol as co-solvent, we evaluated the solubility of UDCA in glycerol and co-solvent/glycerol ratios of 1/4, 1/6, 1/8, and 1/10, for temperatures between 35 and 65 °C (Figure 1). The concentration of UDCA was determined by ^1^H-NMR at 400 MHz after the identification of the adequate signals of UDCA (δ = 0.70; 0.95 ppm) and glycerol (δ = from 4.1 to 4.3 ppm).

The results in Figure 1 show how 2-methylbutan-2-ol favors the solubilization of UDCA in the reaction medium, even at the lowest concentrations. However, the greatest improvement in UDCA solubility occurred at higher co-solvent concentrations, representing 1/6 and 1/4 of the medium (2-methylbutan-2-ol on glycerol ratio). We did not go further with the concentration of co-solvent to maintain a prevailing amount of glycerol in the system, whose stoichiometric prevalence guarantees the shift of the hydrolytic/synthetic balance of the lipase towards its esterification behavior. In addition, the use of organic solvents in enzymatic systems must be carefully managed, and the effects in enzymatic denaturation that occur over time must be evaluated.

### 2.3. Effect of Co-Solvent Concentration on Enzyme Stability

**Figure 2:**
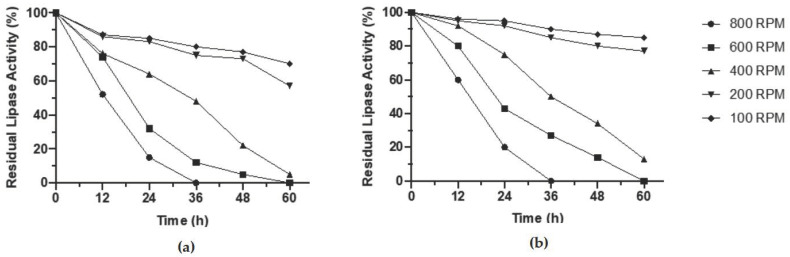
Effect of co-solvent concentration of residual lipase activity of N435. (**a**) Ratio 2-methylbutan-2-ol/glycerol: 1/4. (**b**) Ratio 2-methylbutan-2-ol: 1/6. All the quantifications were performed in triplicate.

Although organic solvents are widely used for enzymatic esterification reactions, some of these could generate phenomena of enzymatic instability. Despite the organic solvent in our experiment representing only a co-solvent helping the solubilization of UDCA in glycerol, its effect on the enzyme stability must be assessed. To evaluate this variable in our SA system, we studied the effects of the two highest concentrations of co-solvent (ratios 1/4 and 1/6) by measuring the residual lipase activity. Residual lipase activity was assessed at 65 °C until 60 h. Furthermore, the effects of the stirring speed were evaluated in a range from 100 to 800 RPM. Indeed, the angular speed plays a key role in enzymatic esterification, ensuring the meeting of substrates; however, too high motion, with the presence of organic solvents, can produce the disruption of the catalysts. The results are shown in Figure 2.

Figure 2 reports the residual enzyme activity in the system with a 1/4 volume ratio of co-solvent (a) and 1/6 of co-solvent (b). It can be observed that in the SA system studied, high stirring speeds produce a complete enzymatic inactivation in 36 h, regardless of the amount of 2-methylbutan-2-ol.

Generally, systems that involve glycerol as a solvent and reagent are used at high stirring speeds [26,37]. These high agitations are allowed by the protective effect of glycerol towards the enzymatic catalyst [30,32]. In our study, the addition of the co-solvent influences the enzymatic stability, especially at high stirring speeds, despite the protective effect of glycerol. In previous studies reported in the literature, the use of high stirring speed was an essential parameter in response to the high density and viscosity of glycerol. In our SA system, the development of a single monophasic medium comprising glycerol and 2-methylbutan-2-ol allows for the enzyme to operate in a less viscous medium where milder stirring is required.

The data show how the concentration of co-solvent has effects on the residual activity of the enzyme, which remains more effective at lower stirring speeds (in particular, 100 and 200 RPM). At 100 and 200 RMP, the residual catalytic capacity remains higher in the SA system with the lower concentration of co-solvent. At 200 RPM in the 1/6 co-solvent media, after 60 h of controlled stirring speed and temperature (65 °C), the enzyme retains 77 ± 0.6% of its catalytic activity. For this reason, the volume ratio of 1/6 2-methylbutan-2-ol to glycerol and the value of 200 RPM were chosen for the subsequent experiments.

### 2.4. Effect of Esterification Parameters

#### 2.4.1. Effect of Enzyme Concentration on Conversion Yield

**Figure 3:**
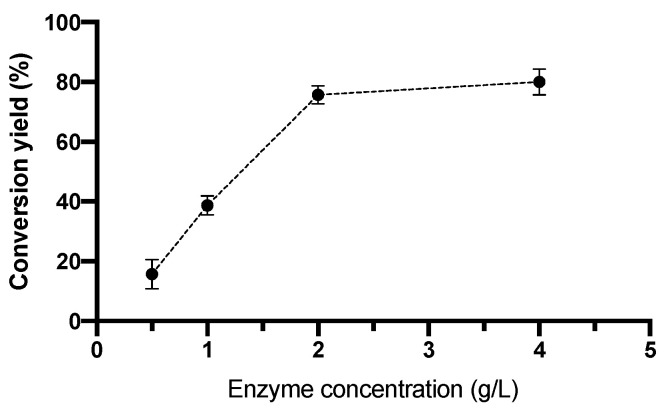
Effect of enzyme concentration on the conversion yield of UDCA-monoglyceride catalyzed by N435. All quantifications were performed in triplicate.

Once the enzymatic esterification media had been defined, we proceeded with the evaluation of the effects of the enzyme concentration on the UDCA-monoglyceride conversion yield. The use of a reverse-phase HPLC allowed us to evaluate the optimal catalyst concentration for our enzymatic system. The results are shown in Figure 3.

To assay the effect of enzyme concentration on conversion yield, we tested four concentrations of N435 in a range between 0.5 and 4 g L^−1^. According to the results shown in Figure 3, we chose the 2 g L^−1^ concentration of biocatalyst as the best for our experimental design. In fact, after the 2 g L^−1^, a small increase in conversion yield is shown, defining the 2 g L^−1^ as the most suitable concentration for this type of enzymatic process.

The curve in Figure 3 shows the hyperbolic trend of the reactions catalyzed by lipase. In fact, depending on the increase in active sites available at the highest concentrations of enzyme, the greater the bond of substrates, and therefore the reaction rate increases.

#### 2.4.2. Effect of Temperature on Conversion Yield

**Figure 4:**
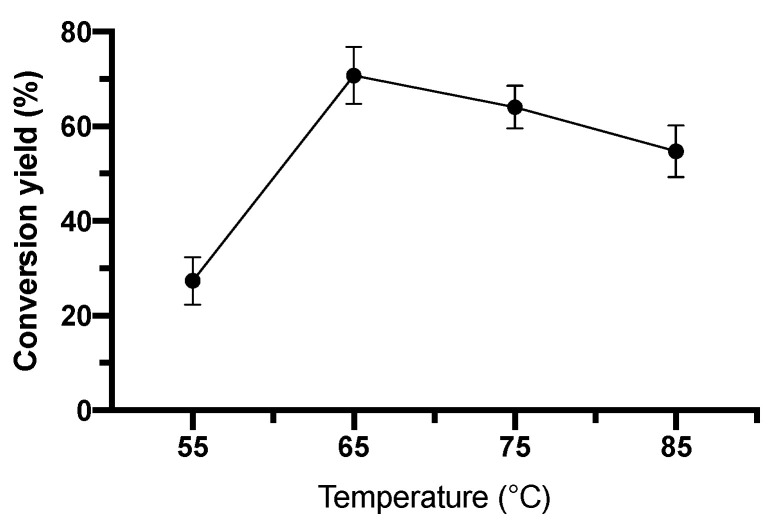
Effect of temperature (°C) on the esterification yield of UDCA-monoglyceride. All quantifications were performed in triplicate.

Temperature is one of the principal parameters affecting the effectiveness of the lipase-catalyzed esterification reaction. Too high temperatures can cause an irreversible decrease in the activity of the enzyme. To assay the effect of temperature on the conversion yield of UDCA-monoglyceride, we tested four temperatures in a range between 55 and 85 °C. The results are given in Figure 4.

At the lowest temperature, the conversion of UDCA-monoglyceride remained limited to about 30%. A net increase in the esterification profile of UDCA-monoglyceride was shown when the temperature is raised from 55 to 65 °C. However, when the temperature was raised, the esterification of UDCA increased until a point upon which no positive changes were detected. At the higher temperature, a decrease in the conversion yield was reported. This phenomenon can be associated with enzyme instability that can cause enzyme inactivation. Thus, we chose 65 °C as the suitable enzyme working temperature.

#### 2.4.3. Effect of Reaction Time on Conversion Yield

**Figure 5:**
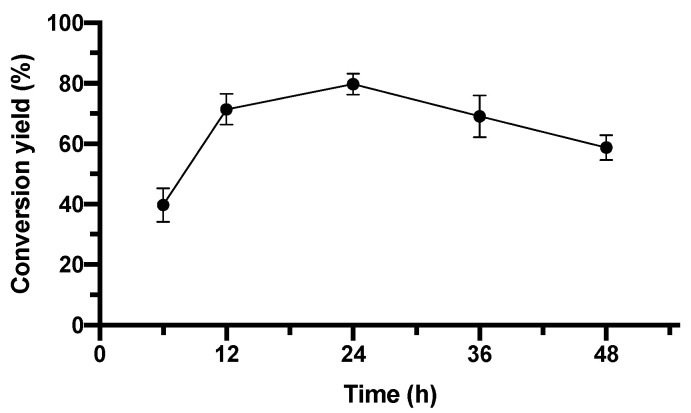
Effect of different reaction times of conversion yield of UDCA-monoglyceride. All quantifications were performed in triplicate.

The effect of reaction time on conversion yield was assessed from 6 to 48 h of esterification reaction. The results are shown in Figure 5.

Ester production was raised with time for up to 24 h of reaction time and then moderately decreased until 48 h, when a conversion yield minor that was generated by a reaction of 12 h was observed. It was probably the case that the effect of the accumulation of water in the system, as a byproduct of the advent of the ester bond, influenced the rate of esterification by activating the lipase in hydrolytic behavior and, thus, hydrolyzing part of the newly formed ester bonds. Due to the abundant quantity of glycerol in the system, this phenomenon occurred rather late (over 36 h), although the use of molecular sieves has not been foreseen.

### 2.5. NMR and uHPLC–MS Characterization of UDCA-Monoglyceride

The attribution of the peaks of the NMR spectra (Figure S1) made it possible to ascertain how the lipase produced a preferential covalent attack towards the primary hydroxyls of glycerol. Furthermore, there are several articles in the literature highlighting how various lipases (including, N435, and porcine pancreatic lipase—PPL) preferentially produce ester bond on the primary hydroxyls of polyols, both short, such as glycerol, and twice as long, as occurs with sorbitol. In fact, various biocatalytic approaches see glycerol (and other polyols, as mentioned, sorbitol) as hydroxyl pendent groups for the production of polyesters. Indeed, the U.S. Food and Drug Administration has proposed glycerol for medical applications, and polymers of glycerol and diacids have generated considerable interest in the development of bioresorbable materials [45]. Riva et al. reported that regioselective acylation of the primary hydroxyl group of monosaccharides with PPL [46]. N435 catalyzed the direct polymerization of sorbitol and adipic acid at the monosubstituted carbon position [47].

**Figure 6:**
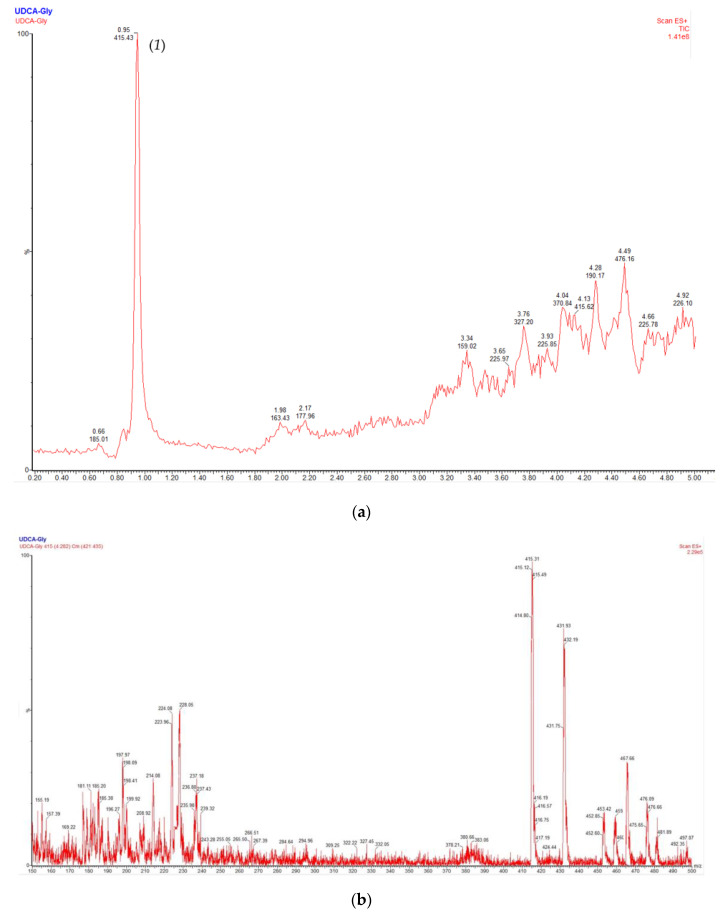
uHPLC–MS analysis of lipase-catalyzed esterification of UDCA and glycerol by N435. (**a**) uHPLC chromatogram, showing the peak of UDCA-monoglyceride (peak No. 1) at 0.95 min; (**b**) ESI+/MS spectrum of the peak No. 1, showing the ester product by electrospray ionization mass spectroscopy.

After performing spectral analyzes of protons and carbons in NMR (Figure S1), we proceeded with the study of the mass of UDCA-monoglyceride. In the uHPLC chromatographic separation, the UDCA ester demonstrated its increased solubility by detecting it at rather short times, at 0.95 min (Figure 6a). We found the aqueous solubility of UDCA-monoglyceride to be six times better (123 mg L^−1^) than the acid form. The sodium salt of UDCA had a vastly higher solubility (200 g L^−1^). However, the esterification of UDCA, creating a prodrug of this active principle, may represent an interesting alternative to the native UDCA, despite its salified form. In fact, although salt is more soluble than UDCA, due to its pka of 4.76, it will easily be found in the dissociated form at acidic stomach pH. At this situation, we believe that the synthesis of a UDCA prodrug can be a viable alternative for the improvement of this active pharmaceutical ingredient [19]. Furthermore, it has been reported that some esters of active principles, reported also for UDCA, not only can raise the water solubility of the drug, but can enhance its biological activity [23,24].

The peak No. 1, derived from the HPLC product separation, was analyzed by electrospray ionization (ESI) to acquire mass spectra. The obtained mass showed the assumed ester product with predicted *m/z* 466. The mass spectrometry results established that the esterification reaction occurred between the carboxylic acid group and the hydroxyl group of glycerol. Figure 1 reports peaks at *m/z* 415 (peak No. 2), 432 (peak No. 3), and 467 (peak No. 4). This fragmentation pattern identifies the UDCA-monoglyceride ester. In fact, masses relative to the molecular weight of the ester without 2 and 3 hydroxyl groups (respectively, *m/z* 432 and *m/z* 415) were observed. Furthermore, the ionized mass value was found (*m/z* 467). The MS analysis showed how UDCA-monoglyceride has a strong tendency to undergo the dehydration effect characteristic of the mass spectroscopy technique, forming the radical carbocation. To the best of our knowledge, this is the first report of the synthesis of the glyceric ester of UDCA. Previously, *C. antarctica* lipase type B has been exploited as a biological catalyst for the production of esters between benzoic acid and glycerol [27]. This SA media esterification is a successful enzymatic route able to minimize the undesired catalytic effect of water in the equilibrium of esterification/hydrolysis of the lipase activity. Indeed, in this system, the short sugar alcohol glycerol acts not only as a reagent, but also as a solvent, creating a net disequilibrium of stoichiometry ratio between alcohol (550 mmol) and acid (3.82 mmol) that improves the esterification reaction; avoids the hydrolysis due to the amount of water produced; and favors the production of the monoglyceride of the acid, limiting the formation of di- and triglycerides. Thus, one of the advantages of this system is the excess of glycerol. The excell of this polyol move the equilibrium of formation of the ester bond towards ester and water products. Moreover, the remaining glycerol could easily be recovered and re-used as solvent and reagent in batch processing or recycled in a continuous process.

## 3. Materials and Methods

### 3.1. Enzyme, Chemicals, and Materials

Novozym^®^ 435 (immobilized lipase B from *C. antarctica*) was kindly gifted by Novozymes A/S (Denmark). Ursodeoxycholic acid (≥99% pure) was purchased from Fluka, Germany. Pure glycerol was obtained from Sigma-Aldrich. Methanol-d4 degree 99.8% was purchased from Sigma-Aldrich. All other solvents were of ACS grade. Silica gel (60 Å, 70–230 mesh, 63–200 µm) was obtained from Sigma-Aldrich. Nuclear magnetic resonance (NMR) spectra of these compounds were recorded with a 400 MHz Varian Gemini spectrometer (Varian, Palo Alto, CA, USA). IR spectra were recorded by a Perkin Elmer FTIR Spectrum 100 infrared spectrometer equipped with ATR using a ZnSe Diamond.

### 3.2. Lipase Activity

The protocol of determination of the initial reaction rate of 4-nitrophenyl butyrate (PNPB) allows for the assaying of the lipase activity. The concentration of the 4-nitrophenol (PNP) produced was studied by UV–VIS spectrophotometry (λ = 348 nm). The analysis was executed in a Jasco V.630 spectrophotometer using 2.5 mL of the stirred solventless or SA system (for each temperature and stirring speed). The substrate (PNPB) solution was prepared in a concentration of 0.4 mM in a 25 mM (pH 7.0) phosphate buffer from a 50 mM stock solution in acetonitrile. The reaction begins with the addition of the appropriate amount of enzyme. Activity is expressed as the initial rate of the hydrolysis reaction referred to as the product p-nitrophenol [27]. The stability of N435 in our systems was measured by withdrawing samples throughout the experiments and subjecting the enzyme to the hydrolytic activity test. The residual activity is determined as the enzymatic activity at a given time over the rate of PNPB hydrolysis reaction at the initial condition.

### 3.3. Effect of the Esterification Parameters

UDCA-monoglyceride ester synthesis was performed with N435 in solventless and solvent-assisted systems in a fixed volume of 50 mL and at various concentrations, times, and stirring speeds. The effect on the esterification yield of four different enzyme concentrations was established in a range from 0.5 to 4 g L^−1^. The effect of the stirring speed was evaluated in the range from 100 to 800 RPM, while the effect of four different temperatures on ester production were examined in a range from 55 to 85 °C. Different effects of reaction time (6–48 h) were studied to find out the best working conditions for UDCA-monoglyceride synthesis.

### 3.4. Thin-Layer Chromatography (TLC)

As a monitoring method, TLC analyses were carried out on the reaction process. A total of 100 µL of the reaction mixtures were diluted in 8 mL of acetone and 1.9 mL of distilled water and analyzed on TLC plates (TLC Silica gel 60, 5 × 10 cm, Merck, Germany) with the elution system ethyl acetate/acetonitrile/acetic acid 60:37:3 (*v/v/v*). In these conditions, the retention factor (R_f_) of the glyceric ester of UDCA was 0.33.

### 3.5. Purification and Spectroscopic Characterization of Glycerol Ester of UDCA

Preliminarily, the whole initial sample was washed 3 times with 10 mL ethyl acetate to remove the excess of unreacted glycerol and extract the ester into the organic solvent. The ethyl acetate was dried with a rotary evaporator, and the purification of the glyceric ester was reached by glass column chromatography. The following eluent solution was used as mobile phase for the silica gel column chromatography: acetate/acetonitrile/acetic acid 60:38.5:1.5 (*v/v/v*). The fractions containing the ester were collected, and the solvent was dried with a rotary evaporator. The physical state of the ester is oily. The rest was analyzed by IR, NMR, and uHPLC. IR: 3366; 2931.54; 2868.05; 1730.39; 1451.41; 1379.69; 1241.21; 1137.93; 1044.82; 931.03; 848.13 cm^−^^1^.

NMR showed the following ^1^H- and ^13^C-NMR spectra (acquired with 400- MHz Varian Gemini spectrometer; Varian, Palo Alto, CA, USA); provided as Figures S2 and S3: ^1^H NMR (400 MHz, CD_3_OD) δ 0.71 (s, 3H, H-18), 0.96 (m, 6H, H-21 and H-19), 1.00–1.67 (m, 18H), 1.78–1.91 (m, 5H), 1.97–2.08 (m, 1H), 2.28–2.41 (m, 2H, H_2_-23), 3.5–3.77 (m, 4H, H-27, H-7), 3.77–3.89 (m, 1H, H-26), 4.03–4.18 (m, 3H), 4.19–4.26 (m, 1H).^13^C NMR, selected peaks (101 MHz CD_3_OD) δ 10.6, 16.9, 19.6, 21.9, 25.9, 27.6, 29, 30, 31.9 (CH_2_-23), 32.2 (CH_2_-22), 34.6, 35.8, 36, 36.6, 38.7, 39.5, 42.5, 41 (C-12), 55.5, 44 (C-13), 56.6 (C-17), 60.8, 63.5 (CH_2_OH-27), 66.5 (CH_2_-25), 71 (CHOH-26), 72.1 (CHOH-3), 174 (C=O-24).

### 3.6. Analytical HPLC Methods

To determine the conversion yield of this preliminary step and all subsequent optimization tests, we calculated the conversion yield by JASCO HPLC modular system equipped with reverse-phase column (Synergi 4 µm Hydro-RP 80 Å − 250 × 4.6 mm), refractive index (model RI-4030), and UV–VIS detector (model UV-4070); 40 °C, mobile phase 50:50 (*v*/*v*) MeCN-phosphate buffer (pH 2.3), 2.0 mL/min. The conversion yield was calculated using the following equation:

(1) $$X=\frac{A_{ester}}{A_{UDCA}-A_{ester}}$$

where $A_{ester}$ means area UDCA-monoglyceride, and $A_{UDCA}$ means area of UDCA. Negative controls of the reaction were prepared without the use of lipase, and all the experiments were conducted in triplicate.

### 3.7. Analytical uHPLC–MS Method

uHPLC–MS analysis was achieved using a Waters Acquity uHPLC provided of a ZQ 2000 ESI mass spectrometry (Waters, Milford, MA, USA) and Mass Link software (Waters, Milford, MA, USA). The 2.6 µm Kinetex 50 mm × 4.6 mm C18 column was selected to perform the analysis. The mobile phases used were water with 0.1% formic acid as solvent A and acetonitrile with 0.1% formic acid as solvent B. The liquid chromatography ran in a gradient condition from 100% H_2_O at t_0_ to 100% acetonitrile at t_5_ (5 min) under a flow rate of 0.3 mL/min. The column operated at a stationary temperature of 40 °C. Temperature, nebulizer pressure, and flow rate of drying gas (N_2_) were 230 °C, 35 psi, and 10 L/min, respectively. The further operation parameters were 1200V for nozzle voltage and 2500 V for the capillary voltage. Mass spectra were tracked in a mass-to-charge (*m/z*) ratio range of 150–500 in positive ion detection mode.

## 4. Conclusions

Ursodeoxycholic acid (UDCA) is a widely used drug limited in bioavailability by its poor water solubility. This work aimed to exploit a lipase-catalyzed reaction in order to obtain effective esterification between ursodeoxycholic acid and glycerol, designing a novel UDCA-monoglyceride derivate with an enhanced hydrophilic profile. The peculiar nature of glycerol, which acted as reagent and solvent, made it possible to investigate both solventless and solvent-assisted reaction environments. The direct enzymatic route has been optimized for biphasic or monophasic media evaluation, enzyme concentration, temperature, stirring speed, and reaction time. The adequate development of the protocol, coupled with the protective effect of glycerol to the enzyme, allowed the system to remain stable and to perfectly recover the reaction product from the medium. ^1^H-NMR, ^13^C-NMR, COSY, HSQC, HMBC, IR, and MS confirmed the N435-catalyzed esterification of UDCA with glycerol. To the best of our knowledge, this is the first time that enzymatic esterification of UDCA-monoglyceride has been proposed.

## Figures and Tables

**Table 1:** Characteristics of the tested organic co-solvents.

Solvent	Boiling Point (°C)	LogP	Solubility in Water	Miscibility with Glycerol
Toluene	110.6	2.43	0.52 mg mL^−1^	Immiscible
2-Methylbutan-2-ol	102.4	0.89	120 mg mL^−1^	Miscible

## Data Availability

Not applicable.

## Supplementary Materials

The following are available online, Figure S1: Attribution Scheme of UDCA-monoglyceride. Figure S2: ^1^H-NMR spectra of UDCA-monoglyceride. Figure S3: ^13^C-NMR of UDCA-monoglyceride. Figure S4: IR spectra of UDCA-monoglyceride. Figure S5: DEPT UDCA-monoglyceride. Figure S6: DEPT Zoom C23 (31.98 ppm; CH2). Figure S7: DEPT Zoom C27 a 63.48 ppm. Figure S8: DEPT Zoom C25 (66.5 ppm; CH2). Figure S9: DEPT Zoom C26 (71.12 ppm, CHOH). Figure S10: COSY UDCA-monoglyceride. Figure S11: COSY Zoom, 2.2 on 1.8. Figure S12: COSY Zoom 4.0 on 4.1. Figure S13: HSQC UDCA-monoglyceride. Figure S14: HSQC Zoom 3.82 on 71.12. Figure S15: HSQC Zoom 4.14 and 4.06 on 66.57. Figure S16: HSQC Zoom 3.55 on 63.4. Figure S17: HSQC Zoom C23 (31.98 ppm; 2.28, 2.39; CH2). Figure S18: HSQC Zoom C25 (66.52 ppm; 4.13, 4.06; CH2) and C26 (67.87 ppm; 4.08; CH). Figure S19: HSQC Zoom 2.28 and 2.39 on 31.9. Figure S20: HMBC UDCA-monoglyceride. Figure S21: HMBC Zoom C=O on 4.14, 4.04, 2.28. Figure S22: HMBC Zoom C27 at 64 ppm on 4.14 e 4.04 and C34 at 66.57 ppm. Figure S23: HMBC 3.55 on C26 at 71 ppm.
