# Supplementary material for: Glyceric Prodrug of Ursodeoxycholic Acid (UDCA): Novozym 435-Catalyzed Synthesis of UDCA-Monoglyceride
Source: Molecules. 2021 Oct 1;26(19):5966. doi: 10.3390/molecules26195966 (PMC8513054; doi:10.3390/molecules26195966)
Supplement: Supplementary file 1 [file molecules-26-05966-s001.zip › molecules-1393536-supplementary.pdf]

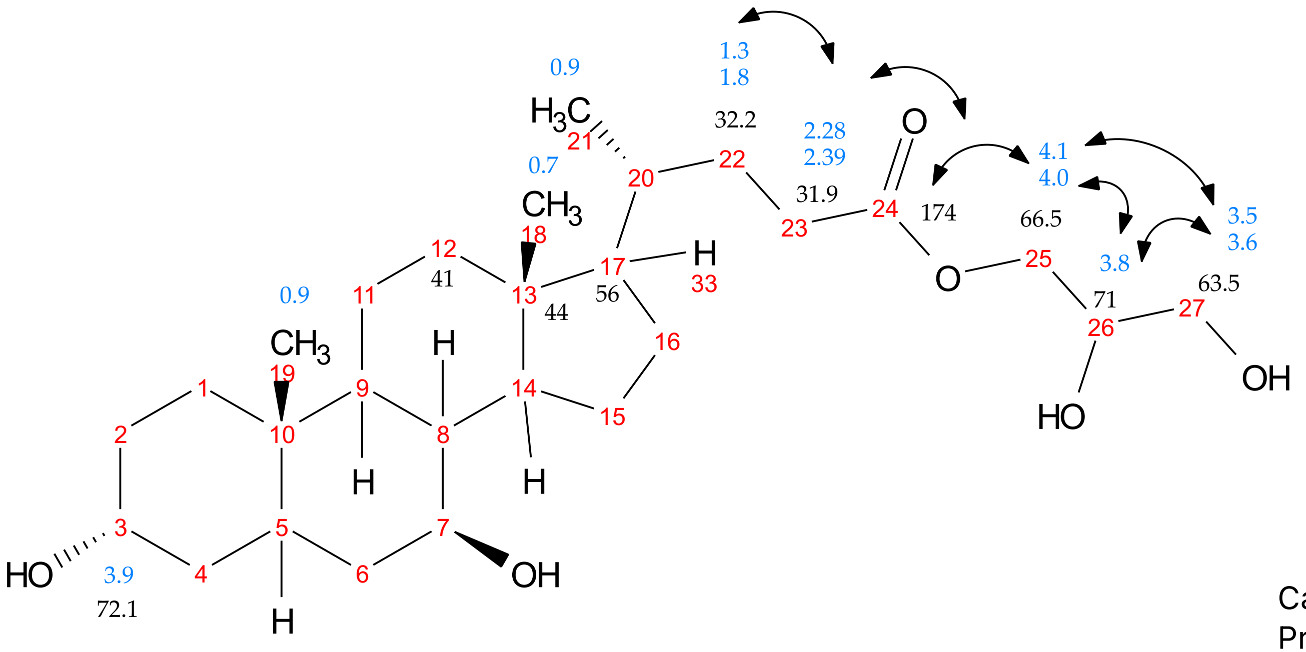

Carbon ppm: Black
Proton ppm: Blue

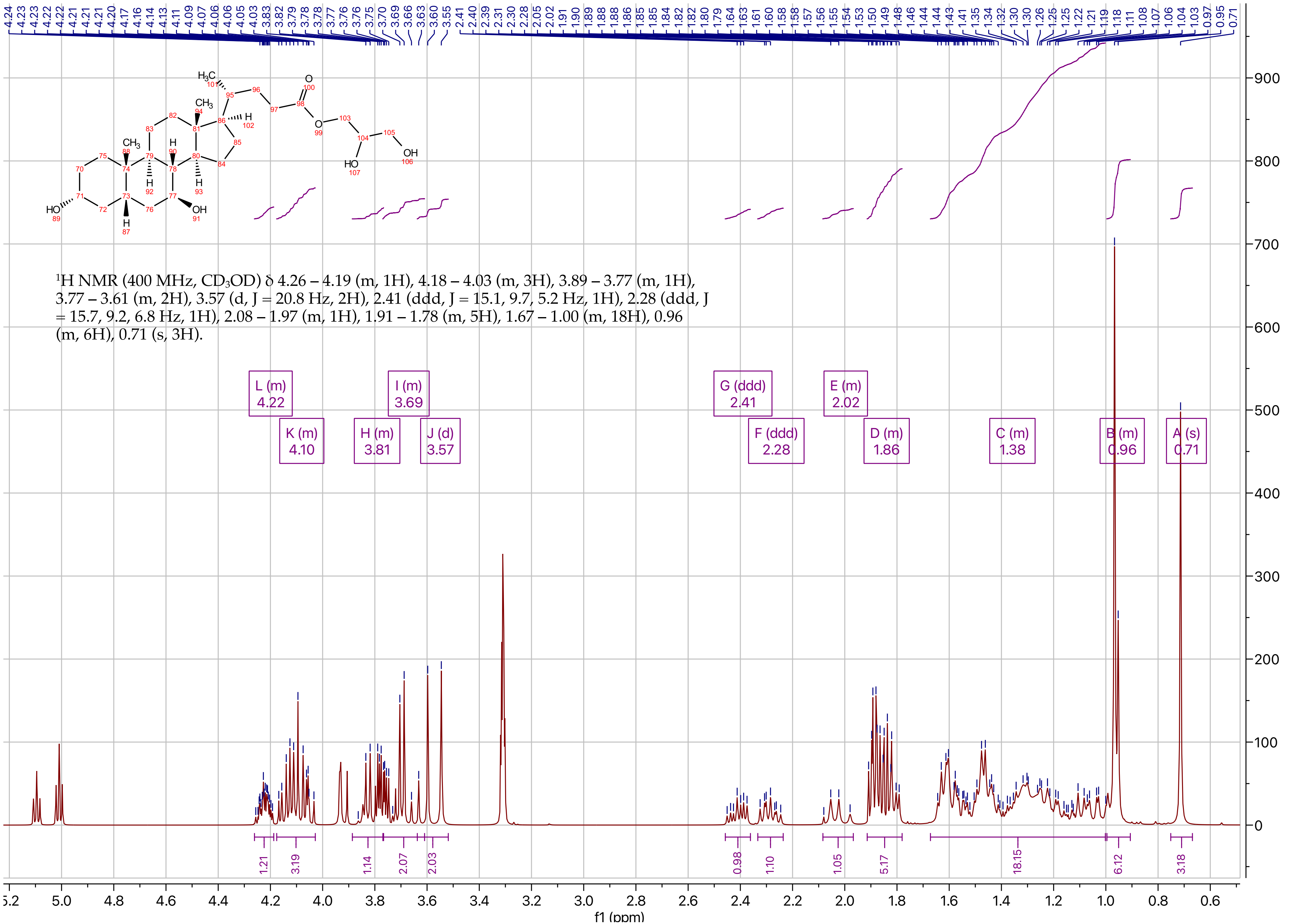

^{1}H NMR (400 MHz, CD_3OD) δ 4.26 – 4.19 (m, 1H), 4.18 – 4.03 (m, 3H), 3.89 – 3.77 (m, 1H), 3.77 – 3.61 (m, 2H), 3.57 (d, J = 20.8 Hz, 2H), 2.41 (ddd, J = 15.1, 9.7, 5.2 Hz, 1H), 2.28 (ddd, J = 15.7, 9.2, 6.8 Hz, 1H), 2.08 – 1.97 (m, 1H), 1.91 – 1.78 (m, 5H), 1.67 – 1.00 (m, 18H), 0.96 (m, 6H), 0.71 (s, 3H).
L (m) 4.22
K (m) 4.10
I (m) 3.69
H (m) 3.81
J (d) 3.57
G (ddd) 2.41
F (ddd) 2.28
E (m) 2.02
D (m) 1.86
C (m) 1.38
B (m) 0.96
A (s) 0.71
1.21
3.19
1.14
2.07
2.03
0.98
1.10
1.05
5.17
18.15
6.12
3.18
f1 (ppm)

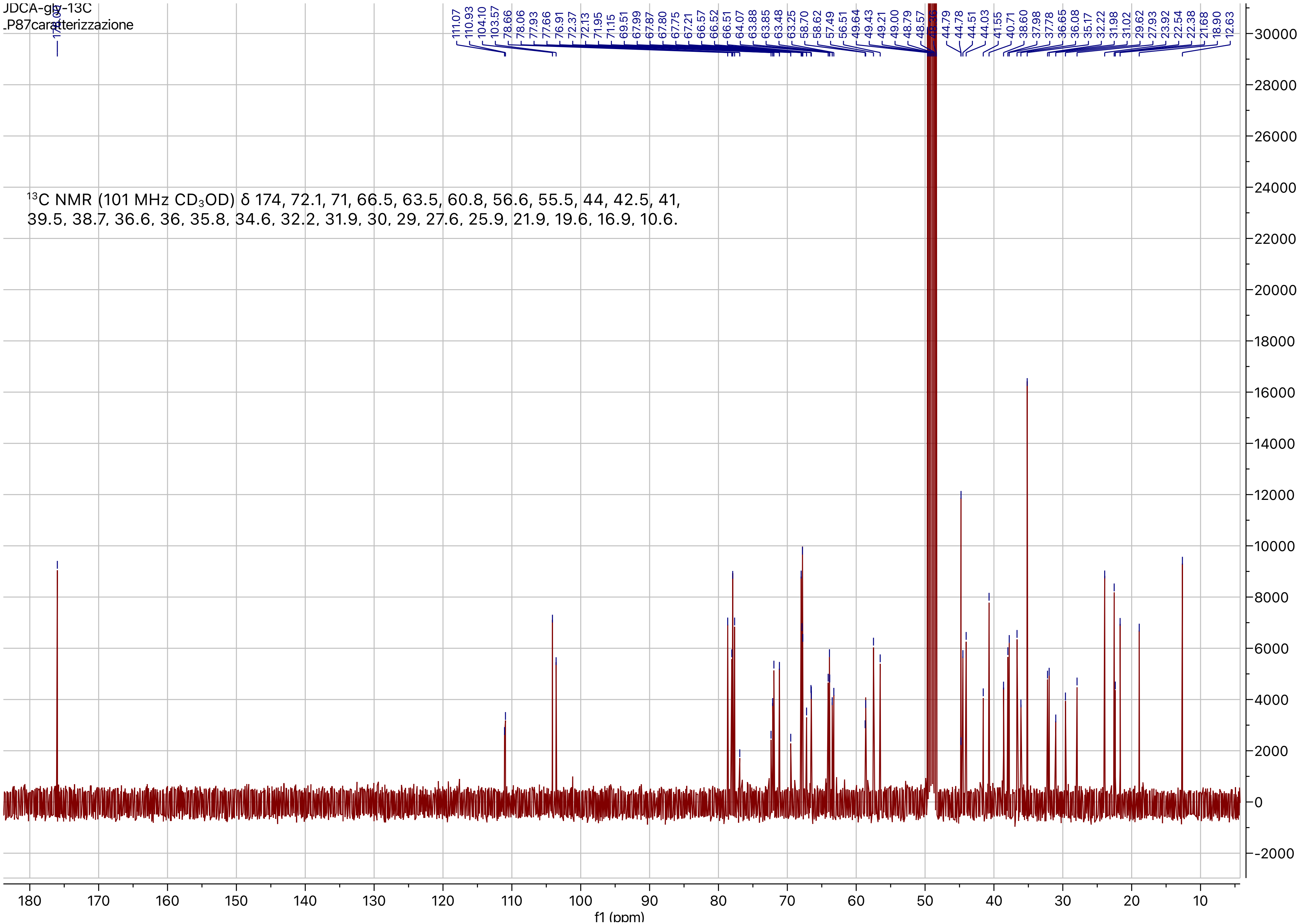
JDCA-gly-13C
LP87caratterizzazione
^{13}C NMR (101 MHz CD_3OD) δ 174, 72.1, 71, 66.5, 63.5, 60.8, 56.6, 55.5, 44, 42.5, 41, 39.5, 38.7, 36.6, 36, 35.8, 34.6, 32.2, 31.9, 30, 29, 27.6, 25.9, 21.9, 19.6, 16.9, 10.6.
111.07 110.93 104.10 103.57 78.66 78.06 77.93 77.66 76.91 72.37 72.13 71.95 71.15 69.51 67.99 67.87 67.80 67.75 67.21 66.57 66.52 66.51 64.07 63.88 63.85 63.48 63.25 58.70 58.62 57.49 56.51 49.64 49.43 49.21 49.00 48.79 48.57 44.79 44.78 44.51 44.03 41.55 40.71 38.60 37.98 37.78 36.65 36.08 35.17 32.22 31.98 31.02 29.62 27.93 23.92 22.54 22.38 21.68 18.90 12.63
f1 (ppm)

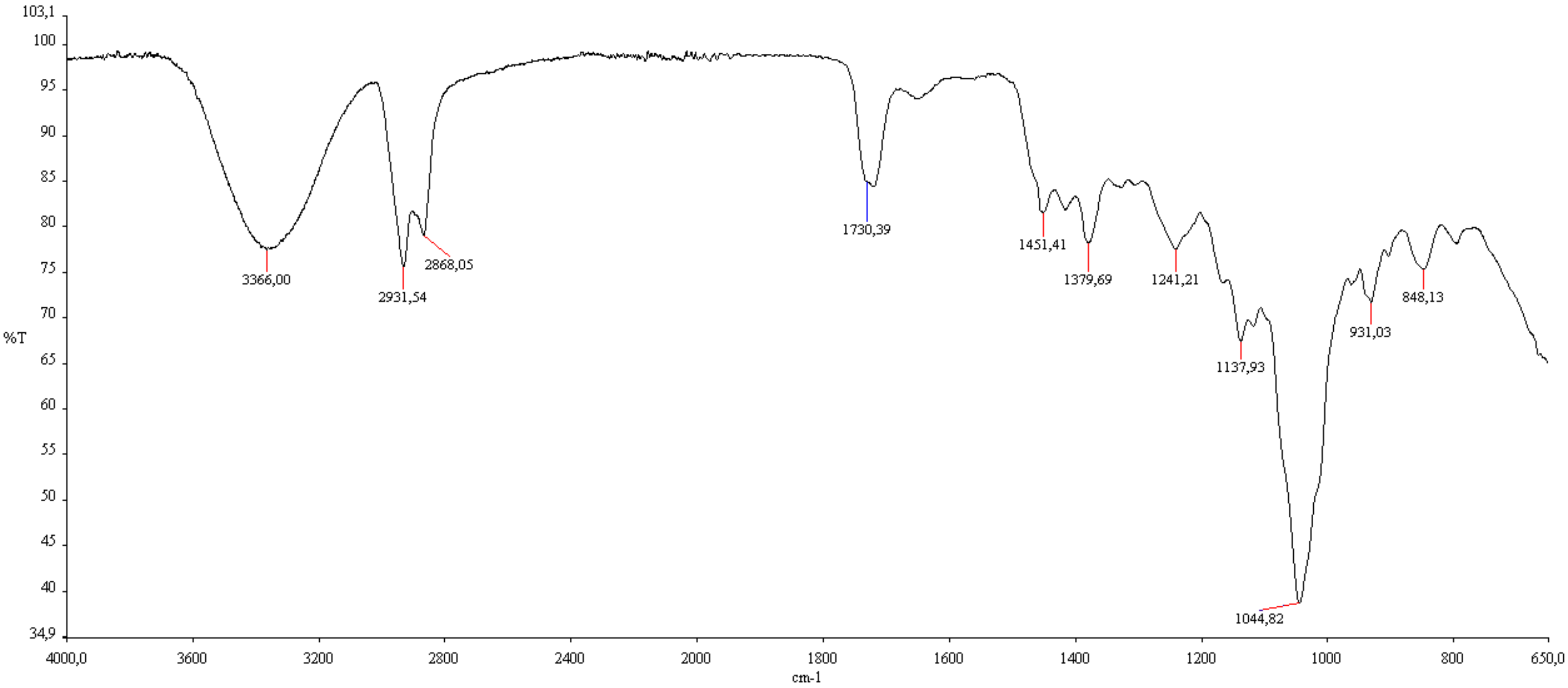

103,1
100
95
90
85
80
75
70
65
60
55
50
45
40
34,9
%T
3366,00
2931,54
2868,05
1730,39
1451,41
1379,69
1241,21
1137,93
1044,82
931,03
848,13
4000,0
3600
3200
2800
2400
2000
1800
1600
1400
1200
1000
800
650,0
cm-1

UDCA-gly-13C
LP87caratterizzazione

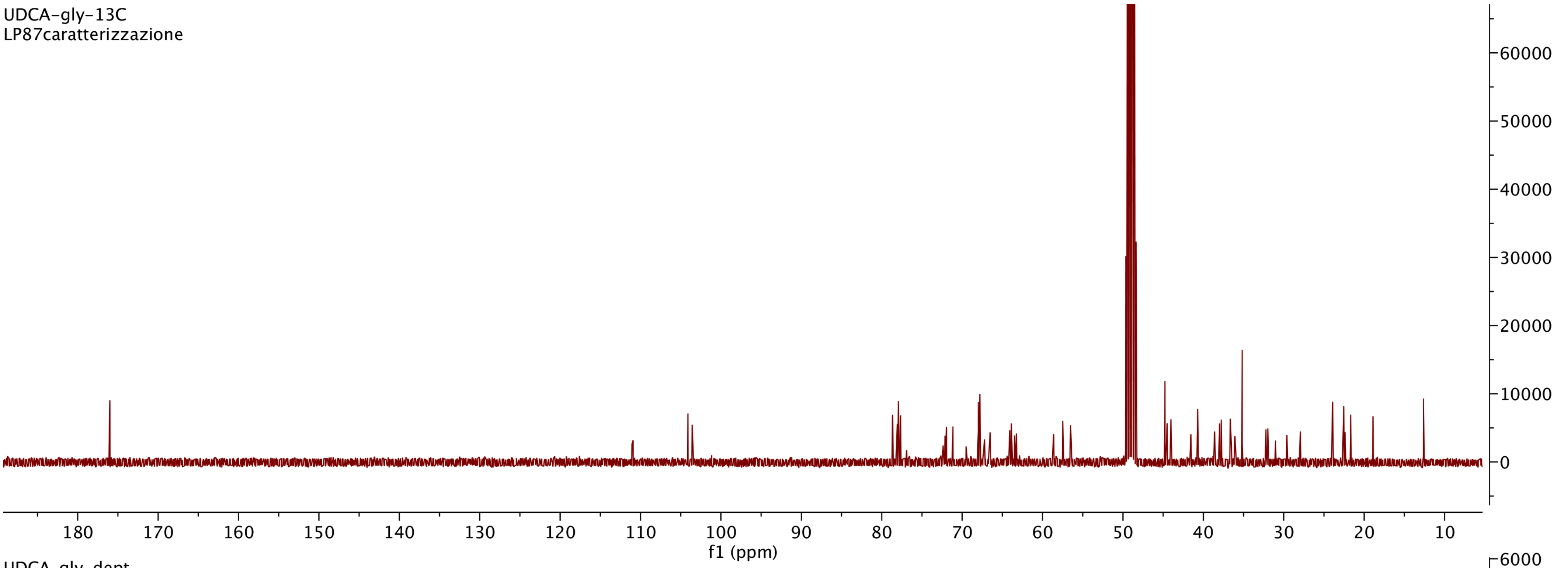

UDCA_gly-dept
UDCA_gly

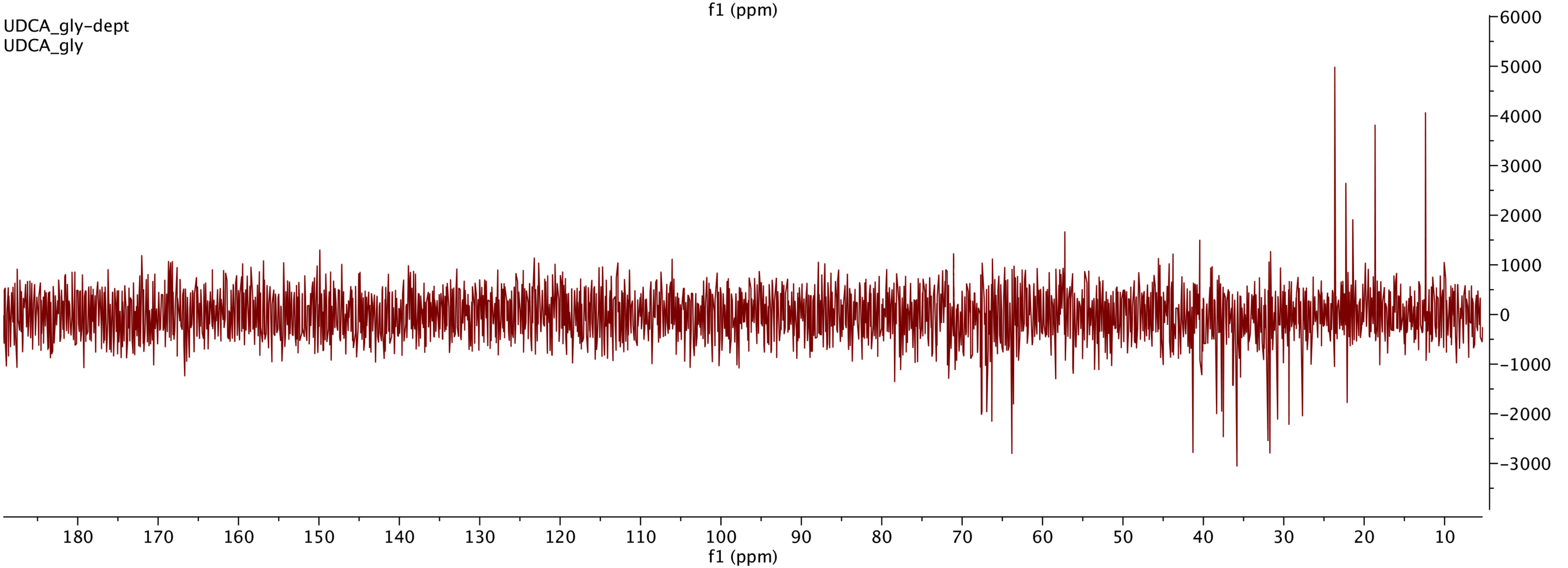

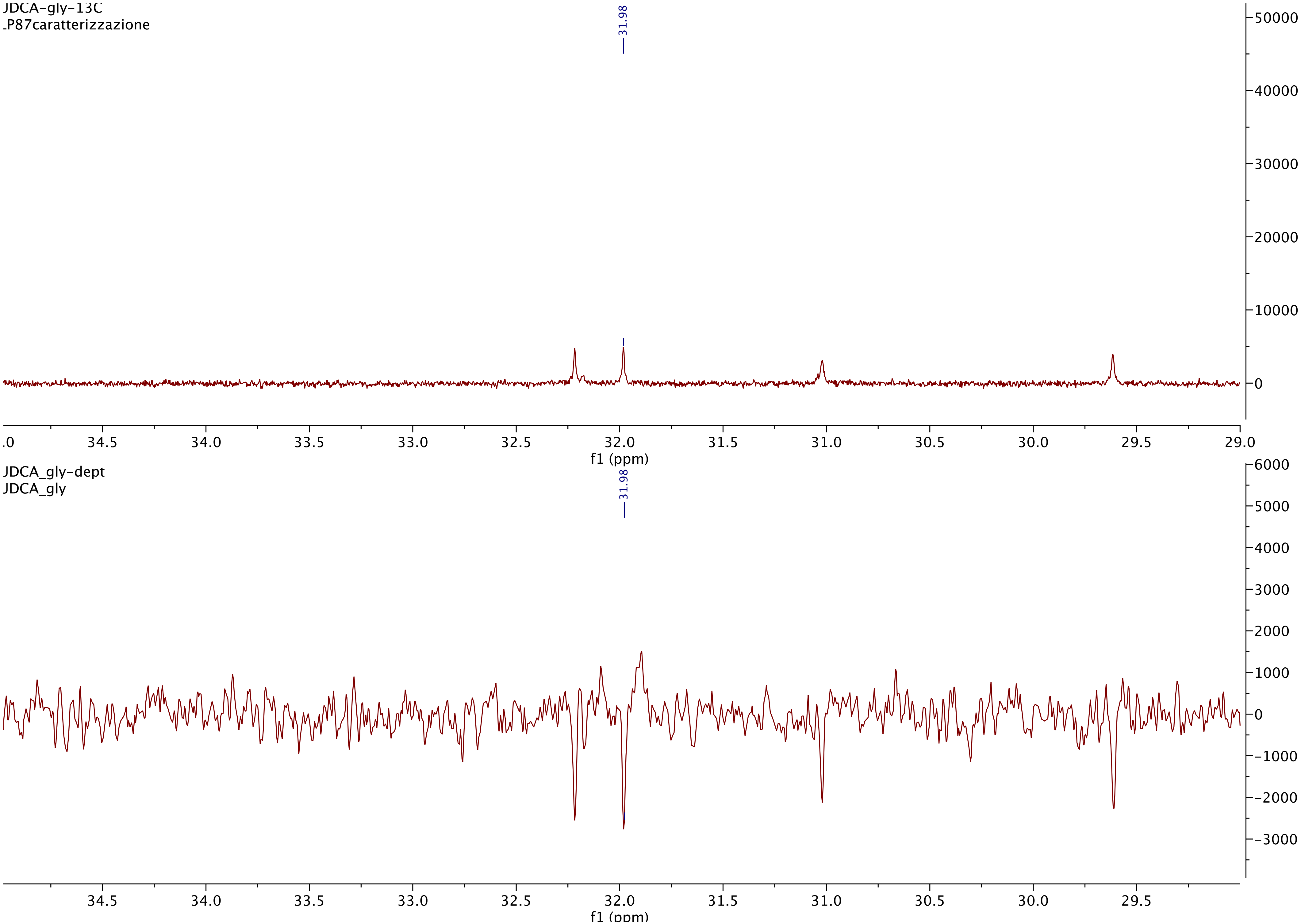

JDCA-gly-13C
.P87caratterizzazione
31.98
f1 (ppm)
JDCA_gly-dept
JDCA_gly
31.98
f1 (ppm)

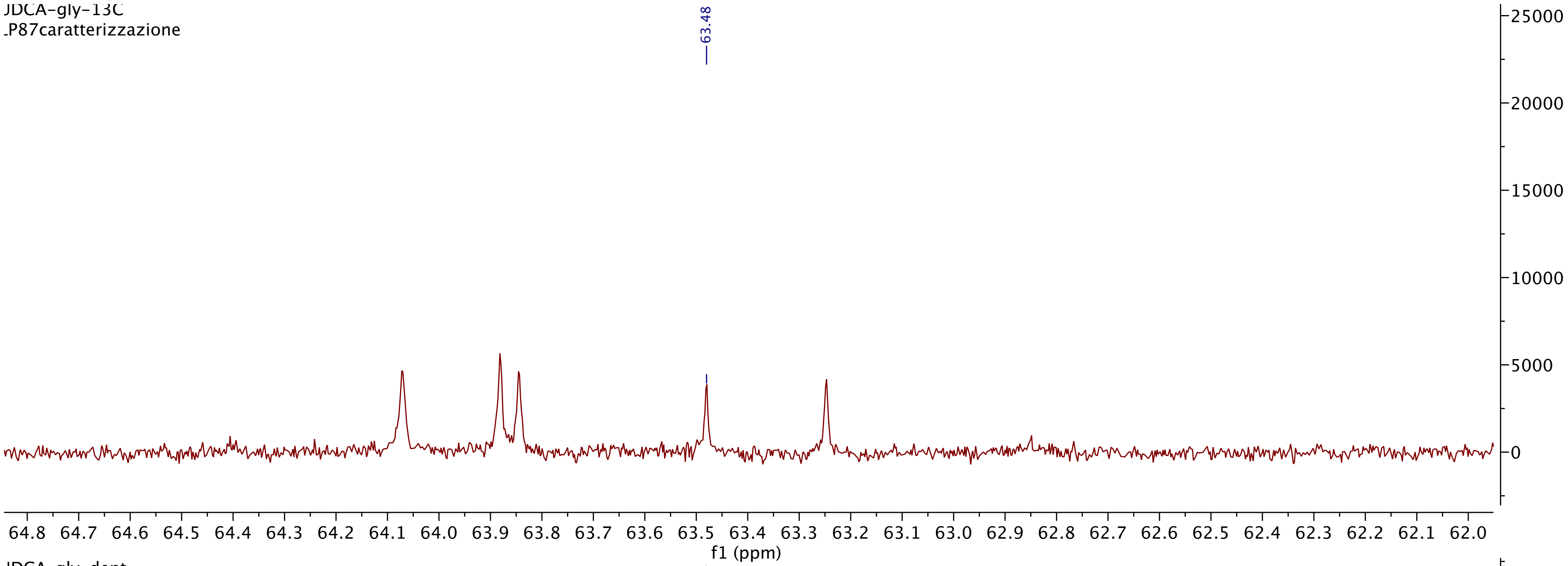

JDCA-gly-13C
.P87caratterizzazione
63.48
25000
20000
15000
10000
5000
0
64.8 64.7 64.6 64.5 64.4 64.3 64.2 64.1 64.0 63.9 63.8 63.7 63.6 63.5 63.4 63.3 63.2 63.1 63.0 62.9 62.8 62.7 62.6 62.5 62.4 62.3 62.2 62.1 62.0
f1 (ppm)

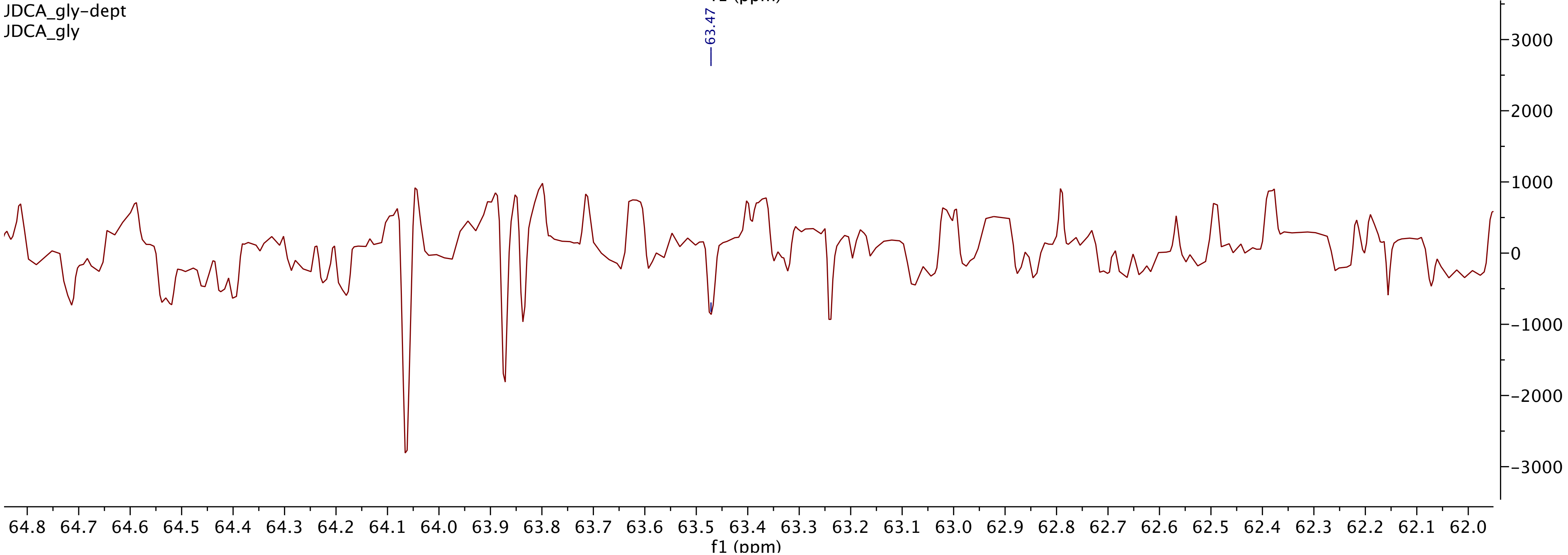

JDCA_gly-dept
JDCA_gly
63.47
3000
2000
1000
0
−1000
−2000
−3000
64.8 64.7 64.6 64.5 64.4 64.3 64.2 64.1 64.0 63.9 63.8 63.7 63.6 63.5 63.4 63.3 63.2 63.1 63.0 62.9 62.8 62.7 62.6 62.5 62.4 62.3 62.2 62.1 62.0
f1 (ppm)

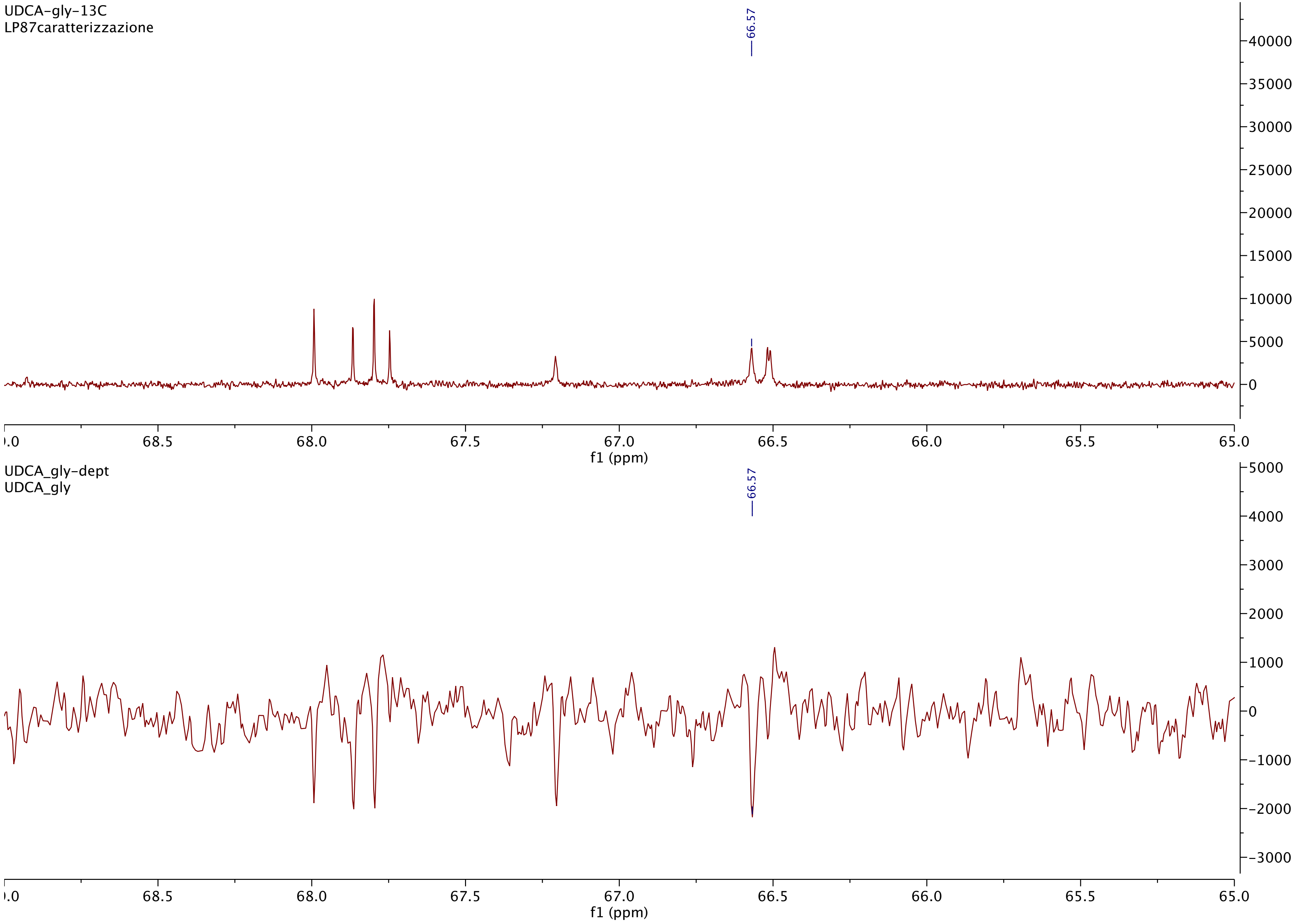
UDCA-gly-13C
LP87caratterizzazione
66.57
40000
35000
30000
25000
20000
15000
10000
5000
0
68.5
68.0
67.5
67.0
66.5
66.0
65.5
65.0
f1 (ppm)
UDCA_gly-dept
UDCA_gly
66.57
5000
4000
3000
2000
1000
0
−1000
−2000
−3000
68.5
68.0
67.5
67.0
66.5
66.0
65.5
65.0
f1 (ppm)

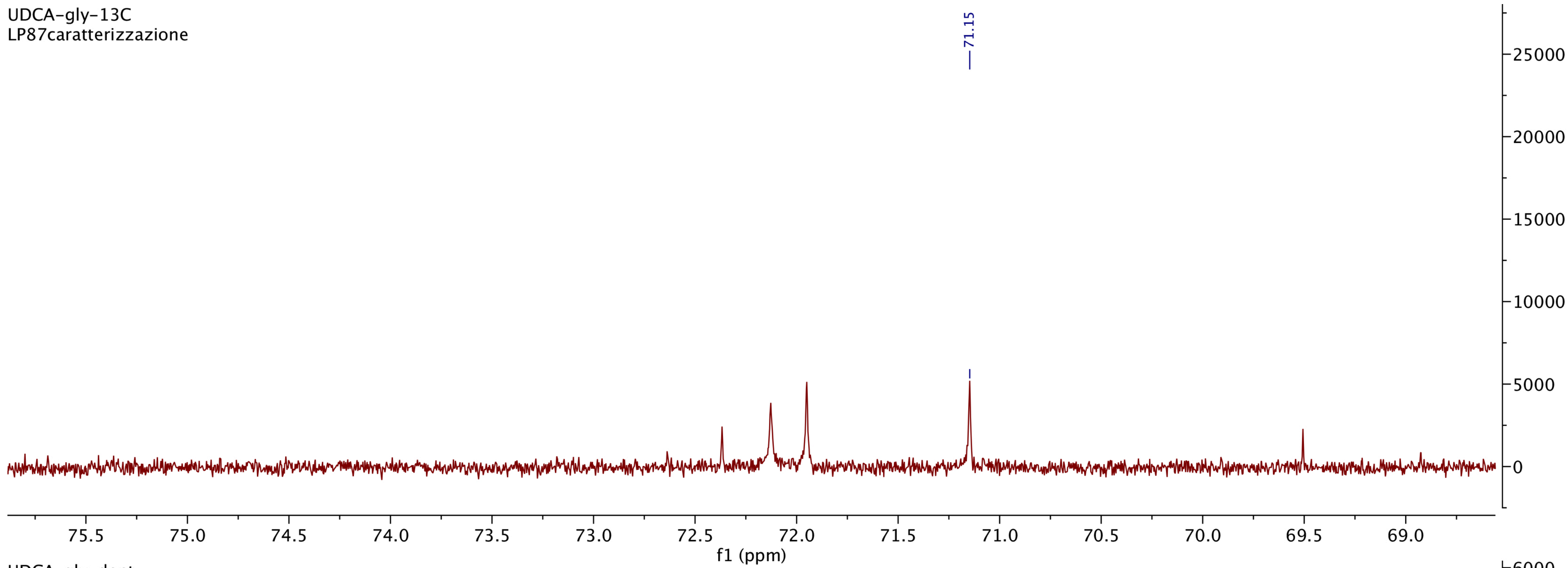

UDCA-gly-13C
LP87caratterizzazione
71.15
f1 (ppm)

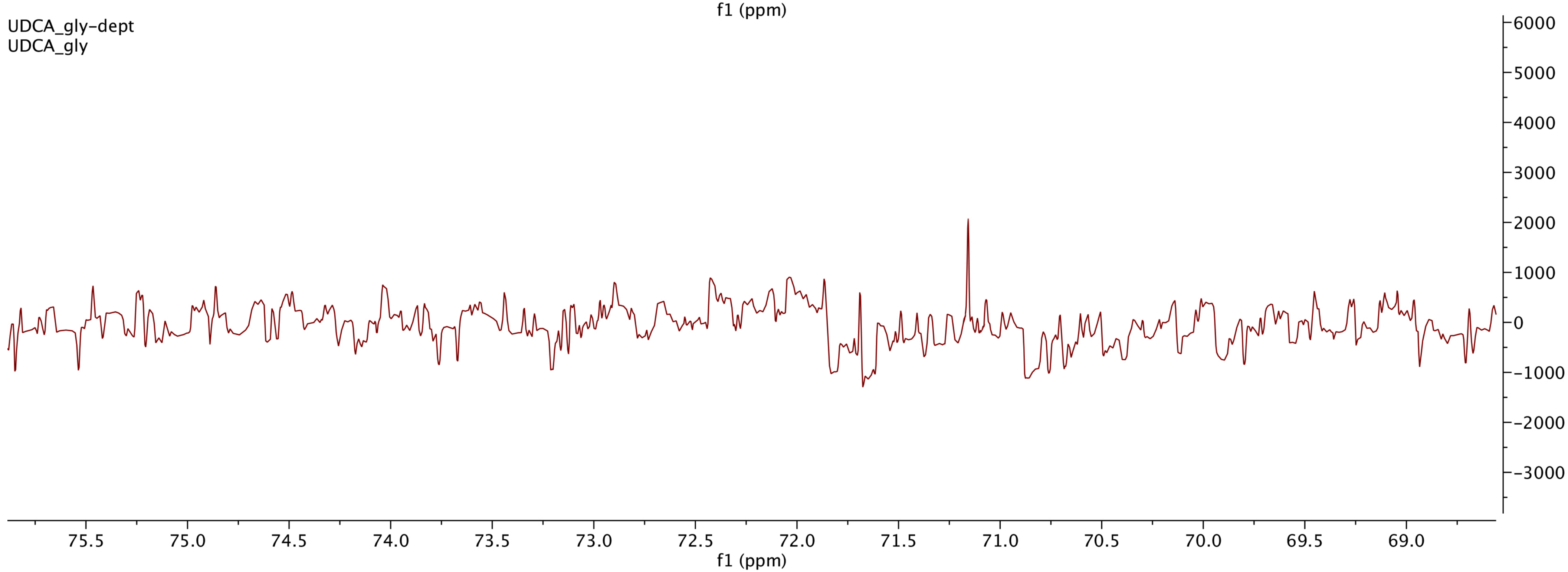

UDCA_gly-dept
UDCA_gly
f1 (ppm)

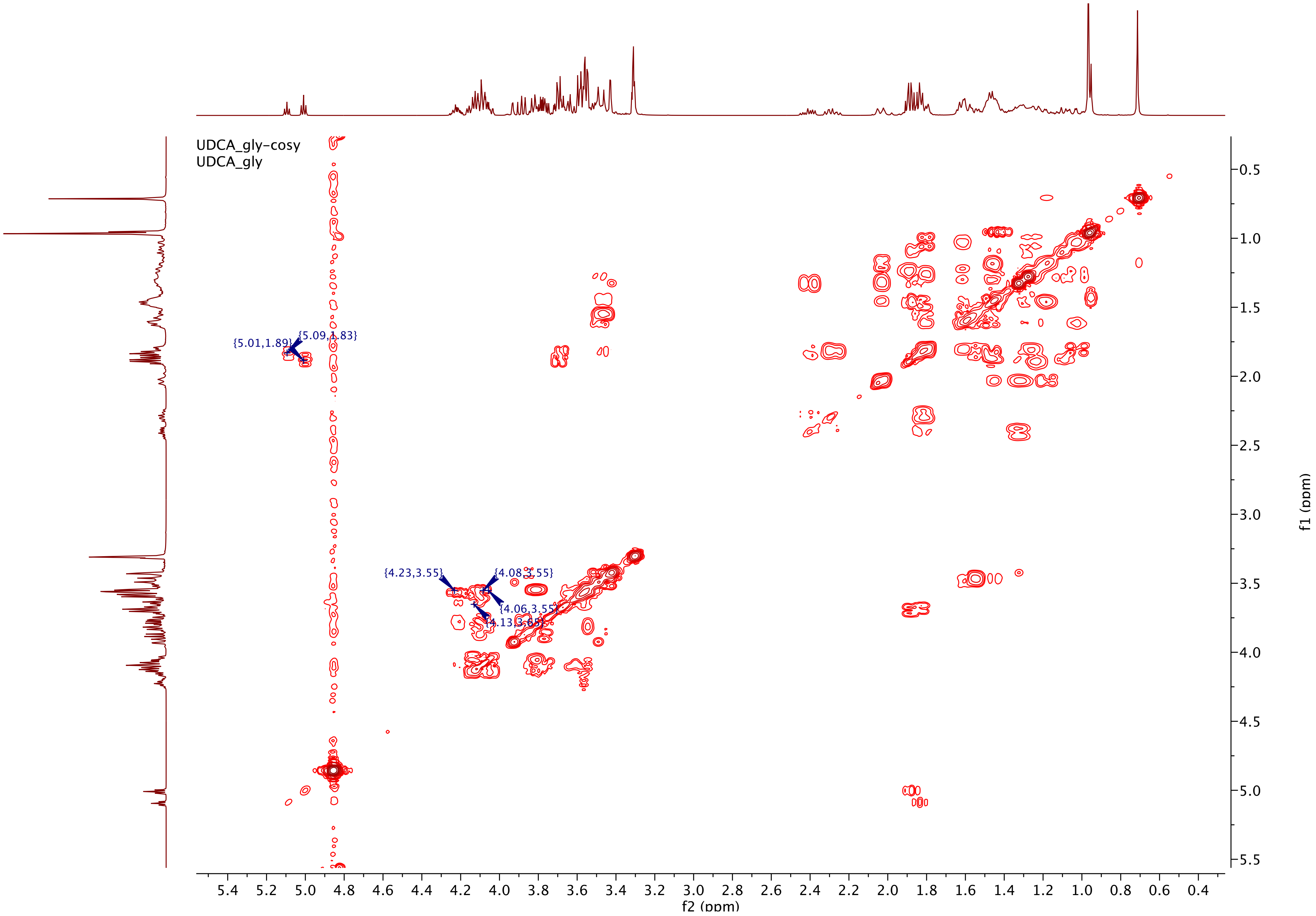

UDCA_gly-cosy
UDCA_gly
{5.01,1.89}
{5.09,1.83}
{4.23,3.55}
{4.08,3.55}
{4.06,3.55}
{4.13,3.65}
f2 (ppm)
f1 (ppm)

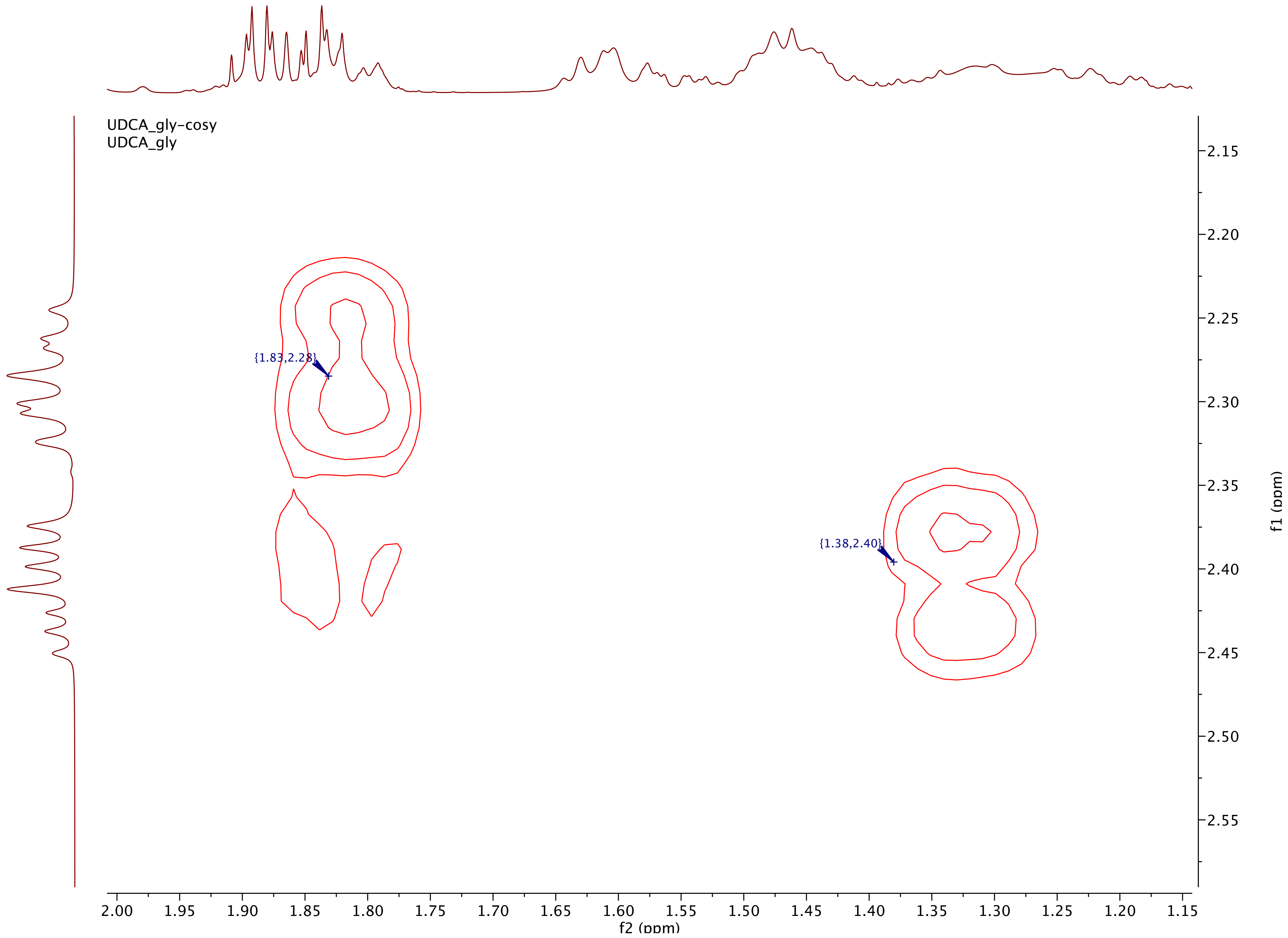
UDCA_gly-cosy
UDCA_gly
{1.83,2.28}
{1.38,2.40}
2.15
2.20
2.25
2.30
2.35
2.40
2.45
2.50
2.55
f1 (ppm)
2.00
1.95
1.90
1.85
1.80
1.75
1.70
1.65
1.60
1.55
1.50
1.45
1.40
1.35
1.30
1.25
1.20
1.15
f2 (ppm)

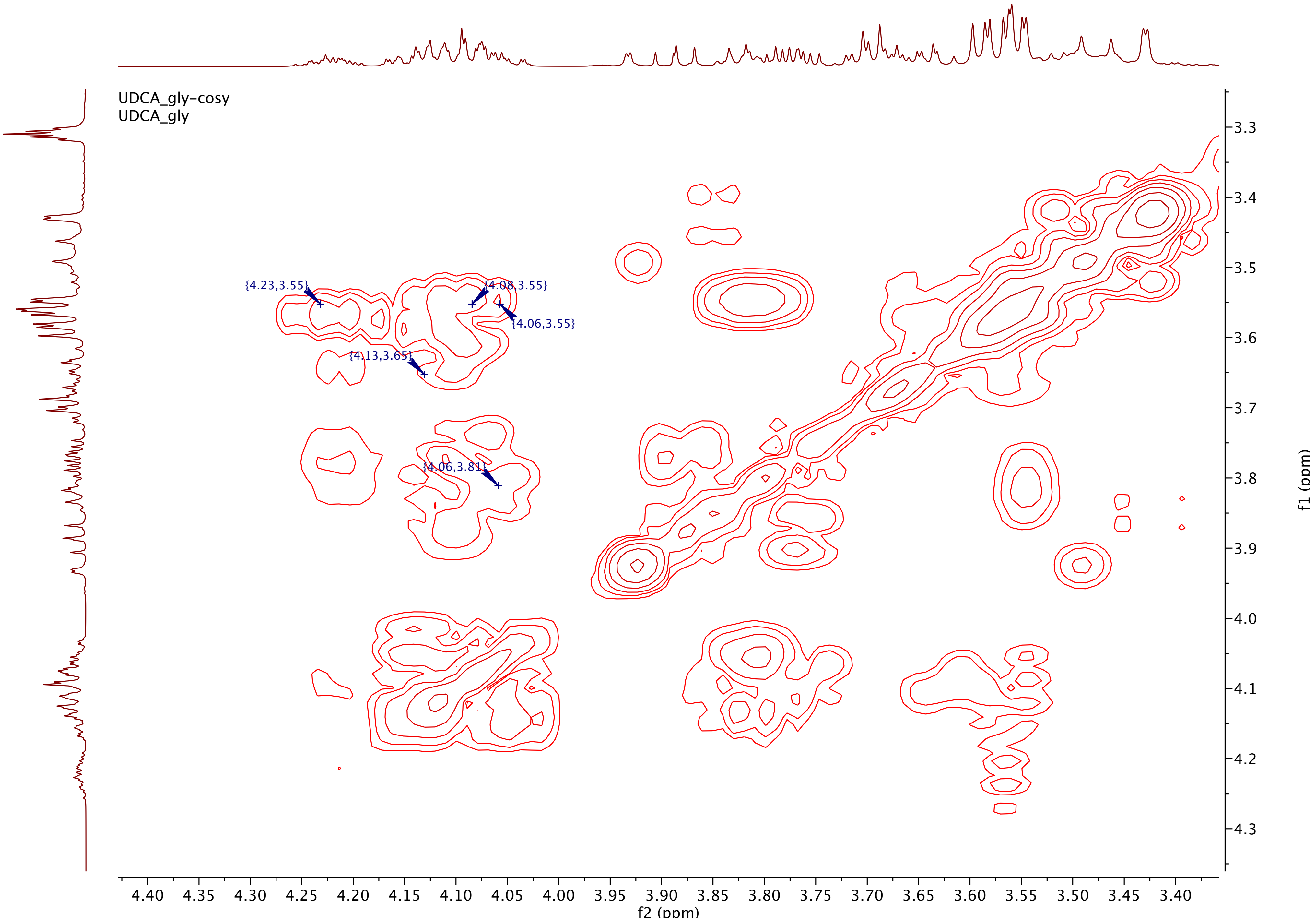
UDCA_gly-cosy
UDCA_gly
{4.23,3.55}
{4.08,3.55}
{4.06,3.55}
{4.13,3.65}
{4.06,3.81}
f2 (ppm)
f1 (ppm)

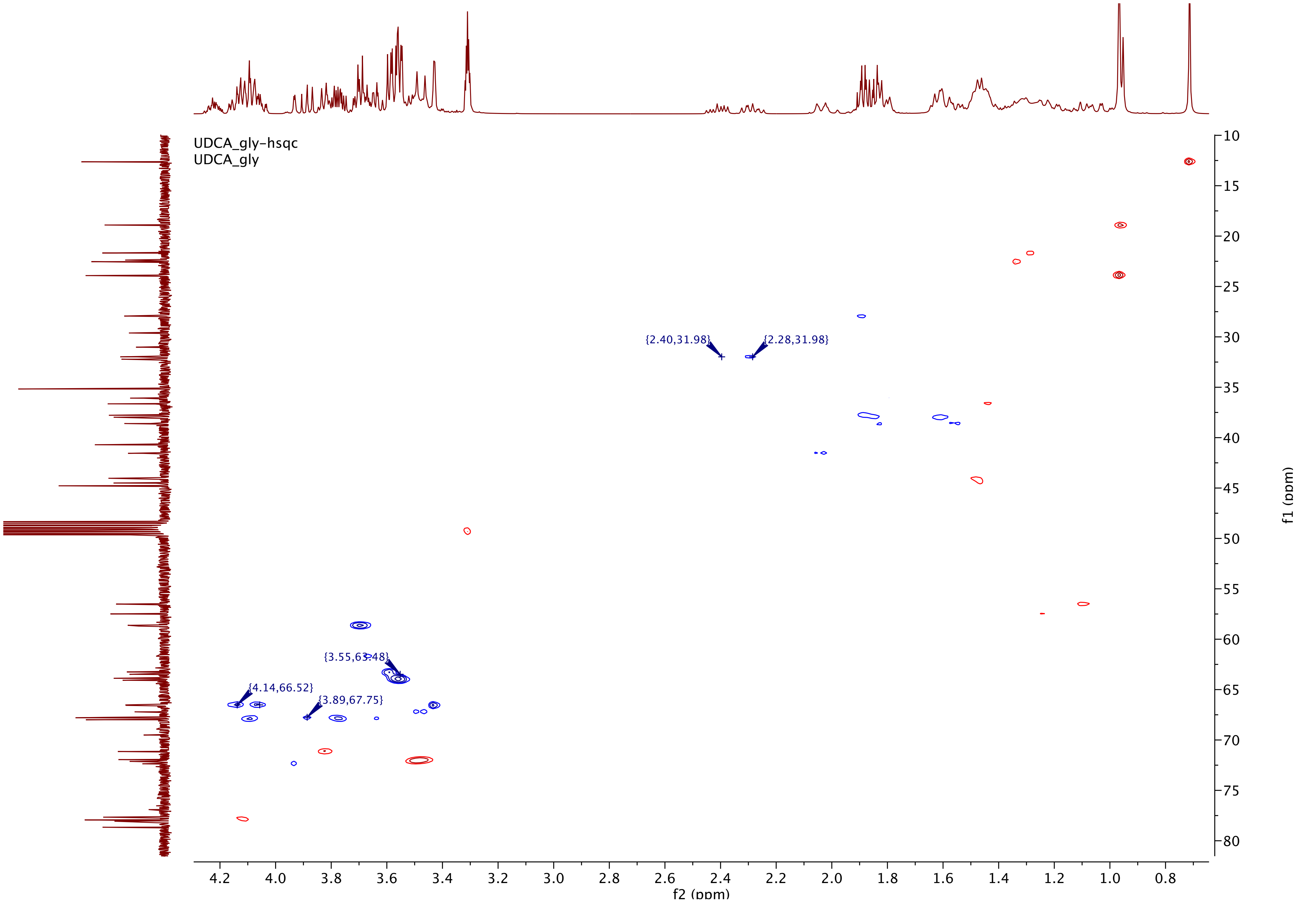

UDCA_gly-hsqc
UDCA_gly
{2.40,31.98}
{2.28,31.98}
{3.55,63.48}
{4.14,66.52}
{3.89,67.75}
f2 (ppm)
f1 (ppm)

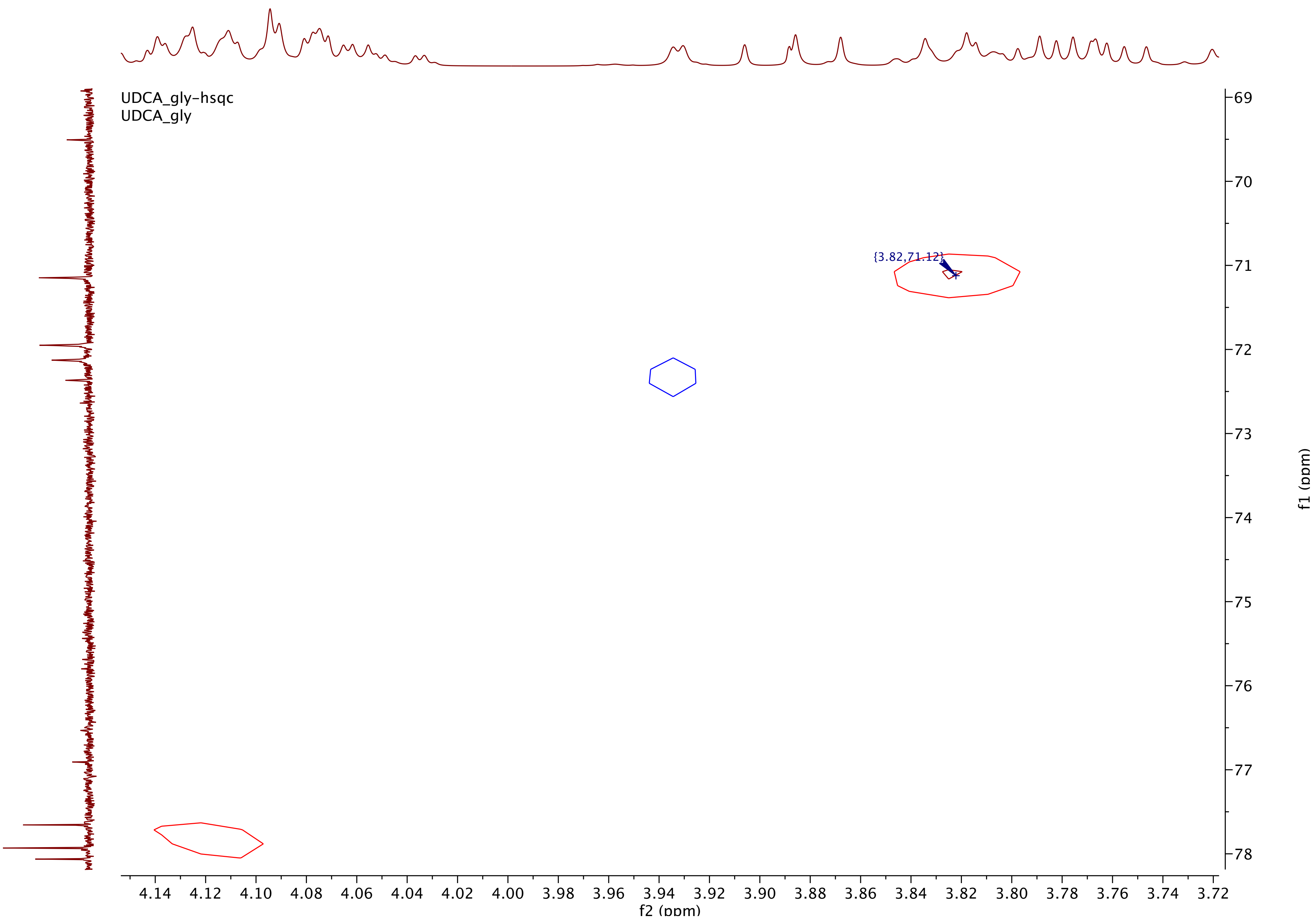
UDCA_gly-hsqc
UDCA_gly
{3.82,71.12}
4.14 4.12 4.10 4.08 4.06 4.04 4.02 4.00 3.98 3.96 3.94 3.92 3.90 3.88 3.86 3.84 3.82 3.80 3.78 3.76 3.74 3.72
f2 (ppm)
69 70 71 72 73 74 75 76 77 78
f1 (ppm)

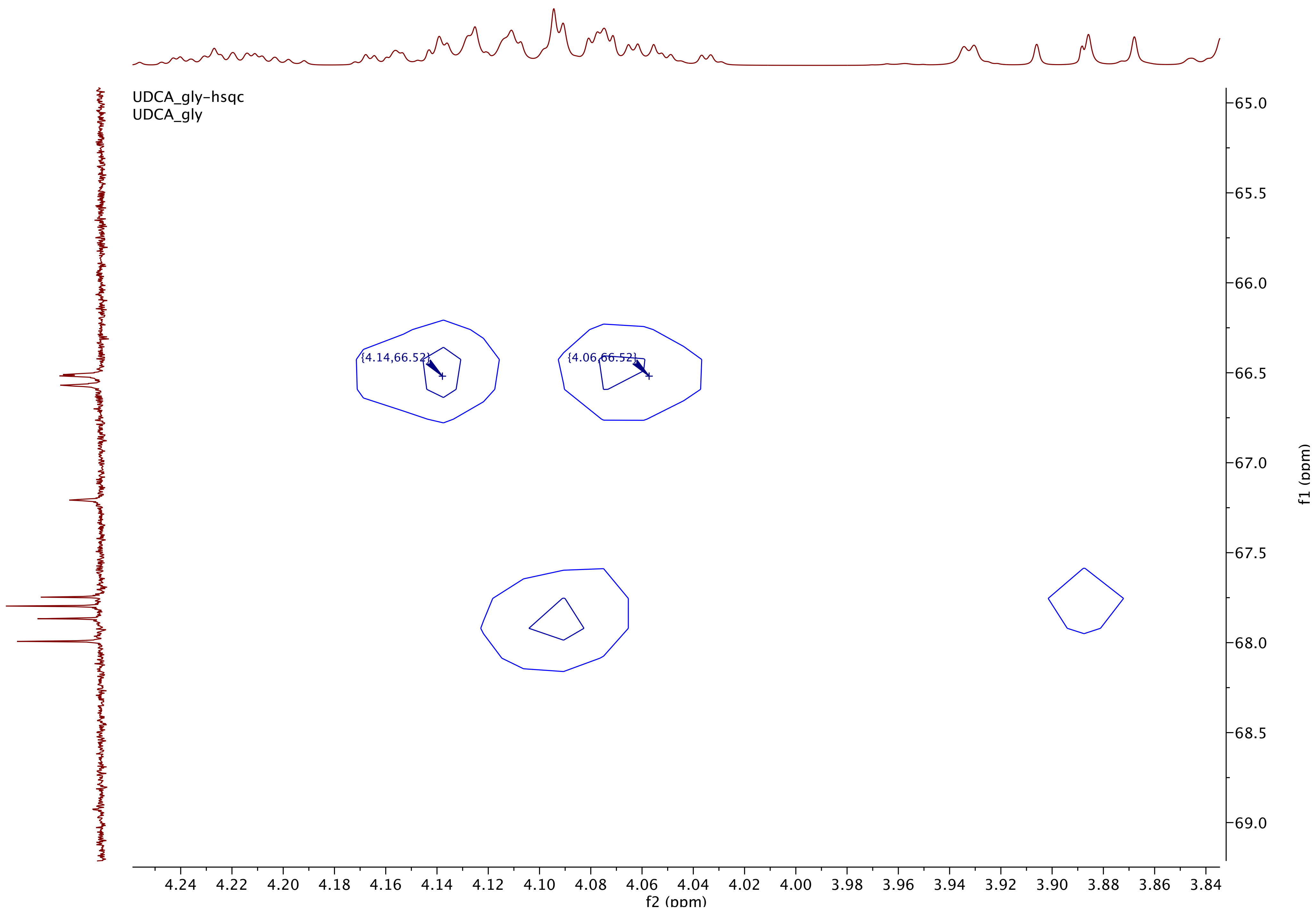
UDCA_gly-hsqc
UDCA_gly
{4.14,66.52}
{4.06,66.52}
f1 (ppm)
f2 (ppm)

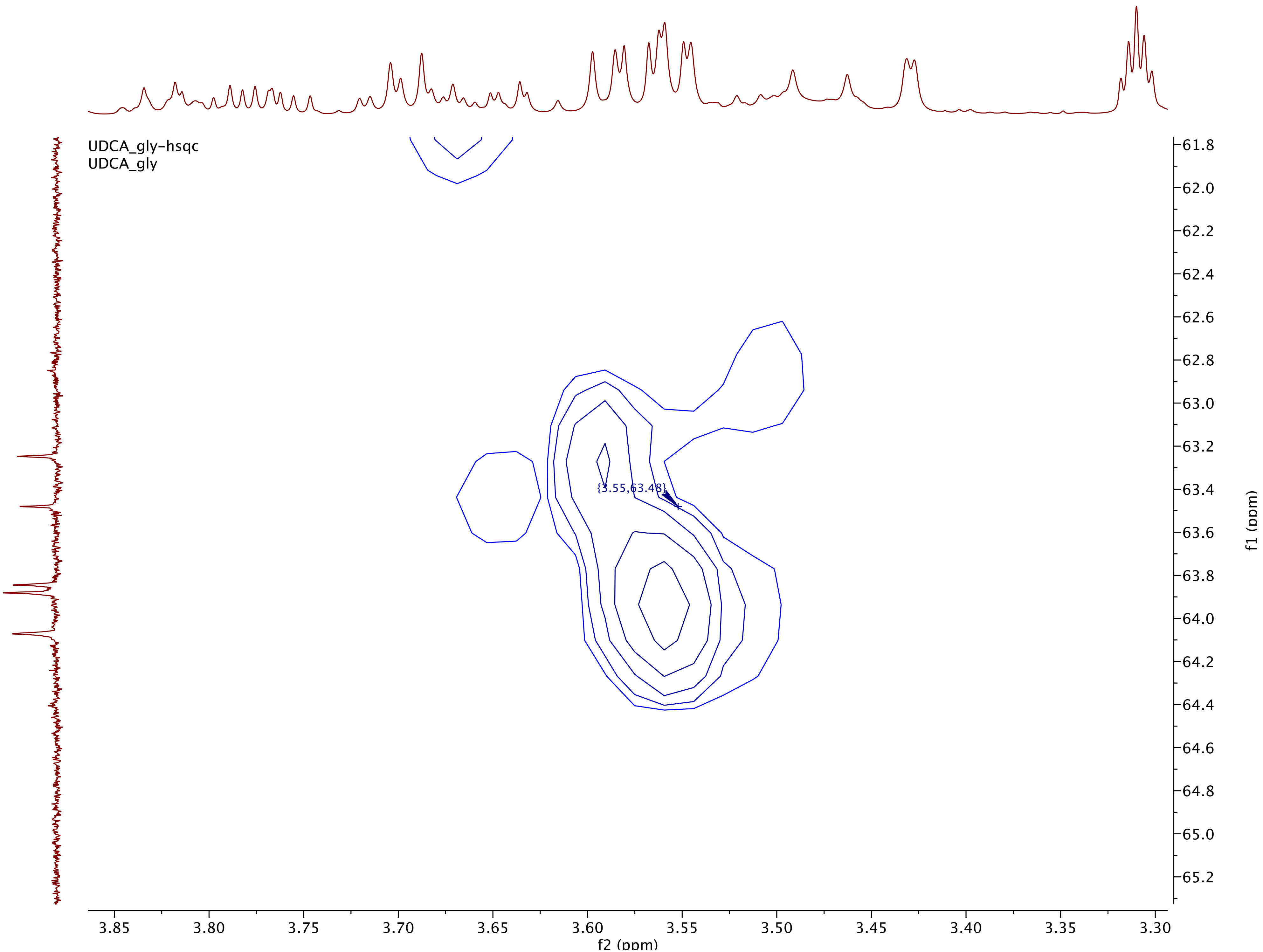
UDCA_gly-hsqc
UDCA_gly
{3.55,63.48}
f2 (ppm)
f1 (ppm)
3.85
3.80
3.75
3.70
3.65
3.60
3.55
3.50
3.45
3.40
3.35
3.30
61.8
62.0
62.2
62.4
62.6
62.8
63.0
63.2
63.4
63.6
63.8
64.0
64.2
64.4
64.6
64.8
65.0
65.2

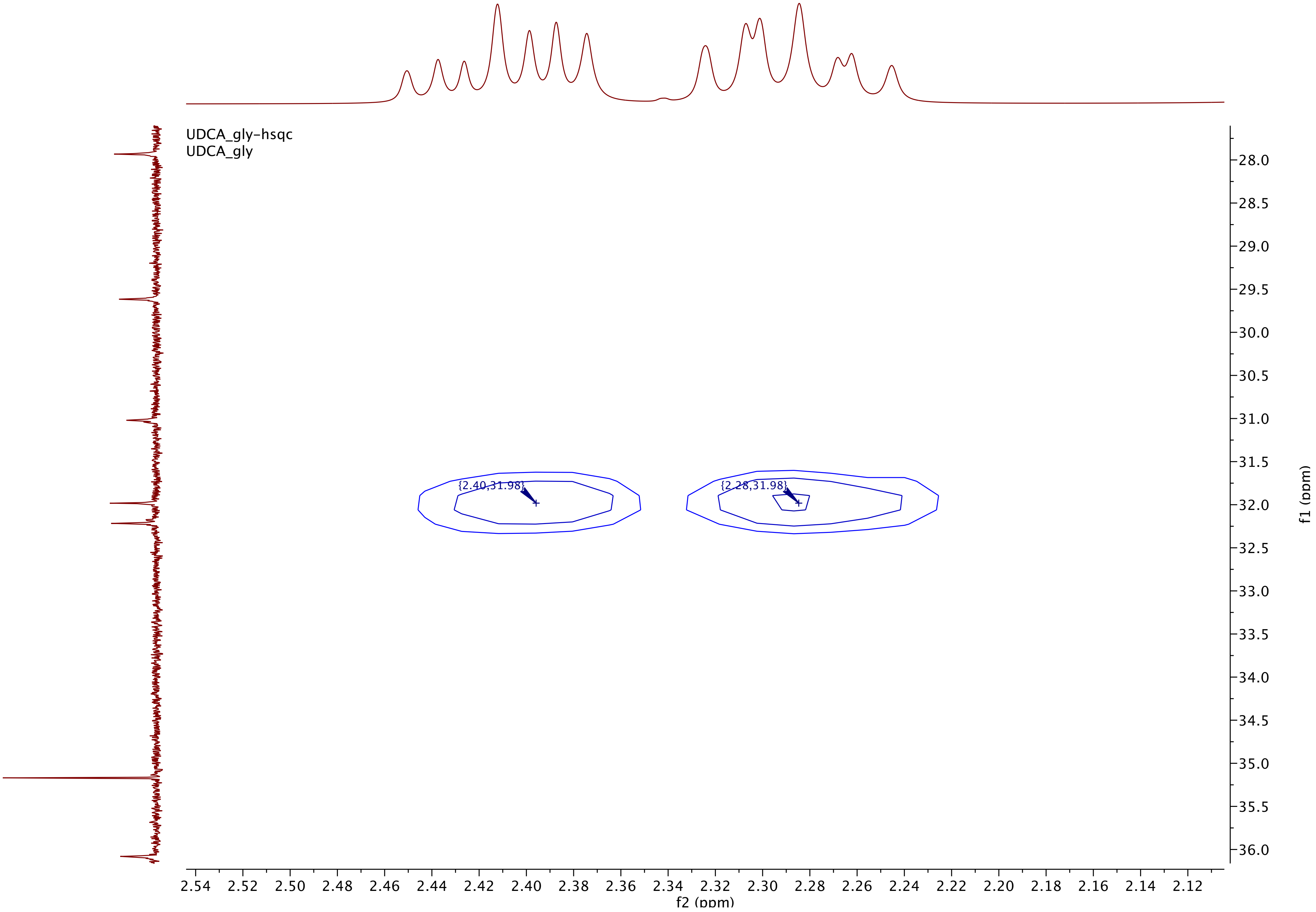
UDCA_gly-hsqc
UDCA_gly
{2.40,31.98}
{2.28,31.98}
f1 (ppm)
f2 (ppm)

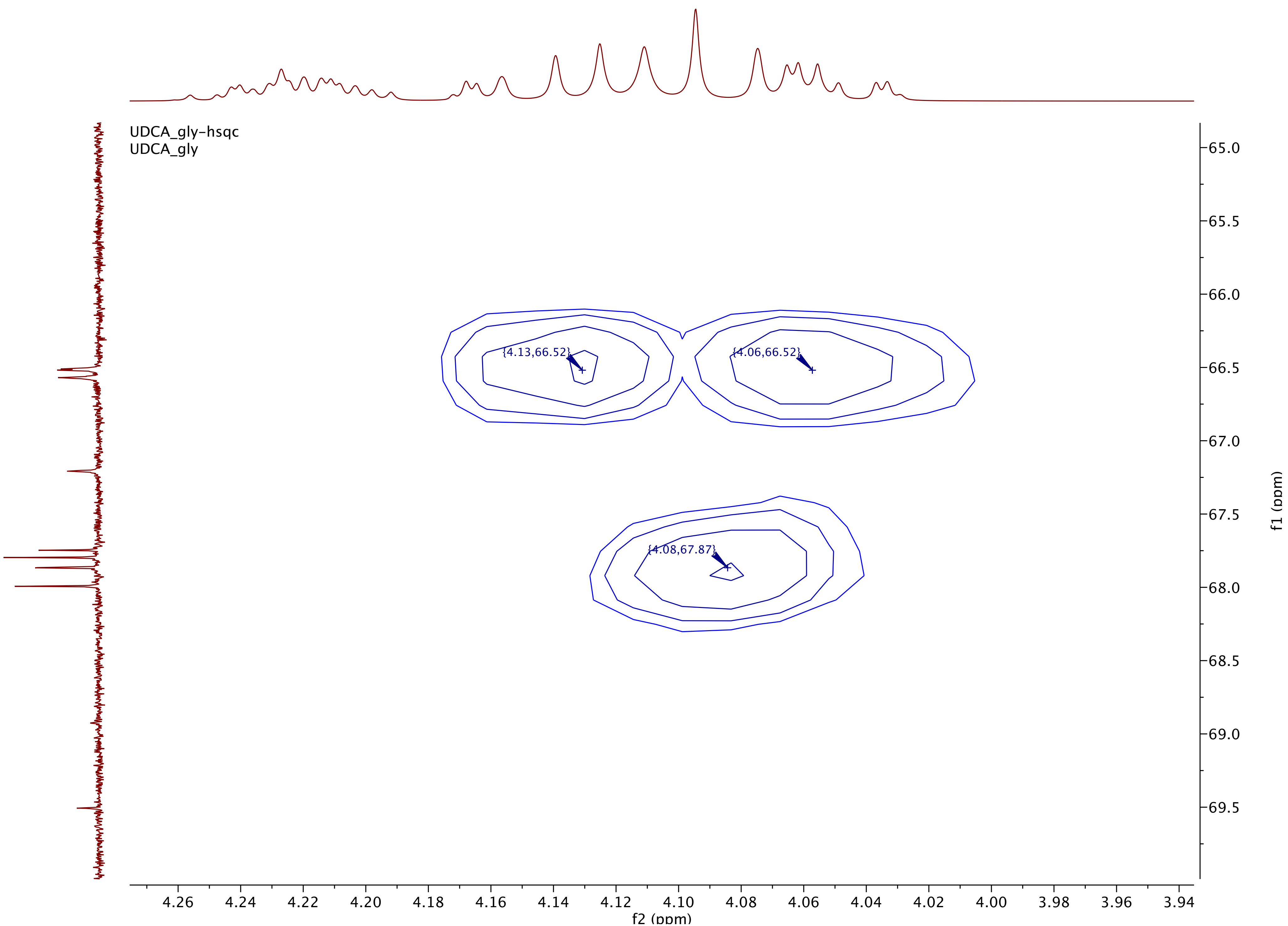
UDCA_gly-hsqc
UDCA_gly
{4.13,66.52}
{4.06,66.52}
{4.08,67.87}
f2 (ppm)
f1 (ppm)

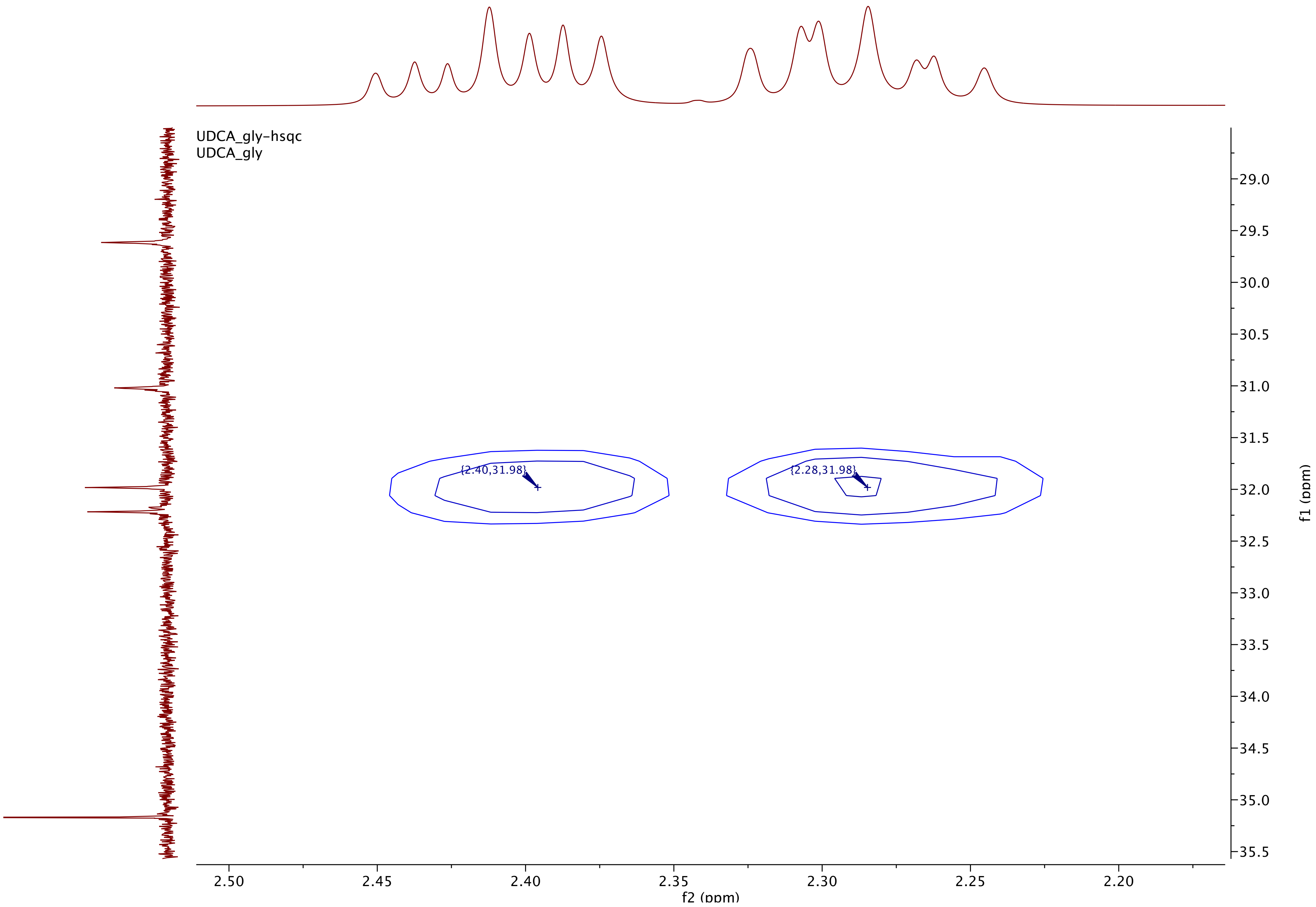
UDCA_gly-hsqc
UDCA_gly
{2.40,31.98}
{2.28,31.98}
f1 (ppm)
f2 (ppm)

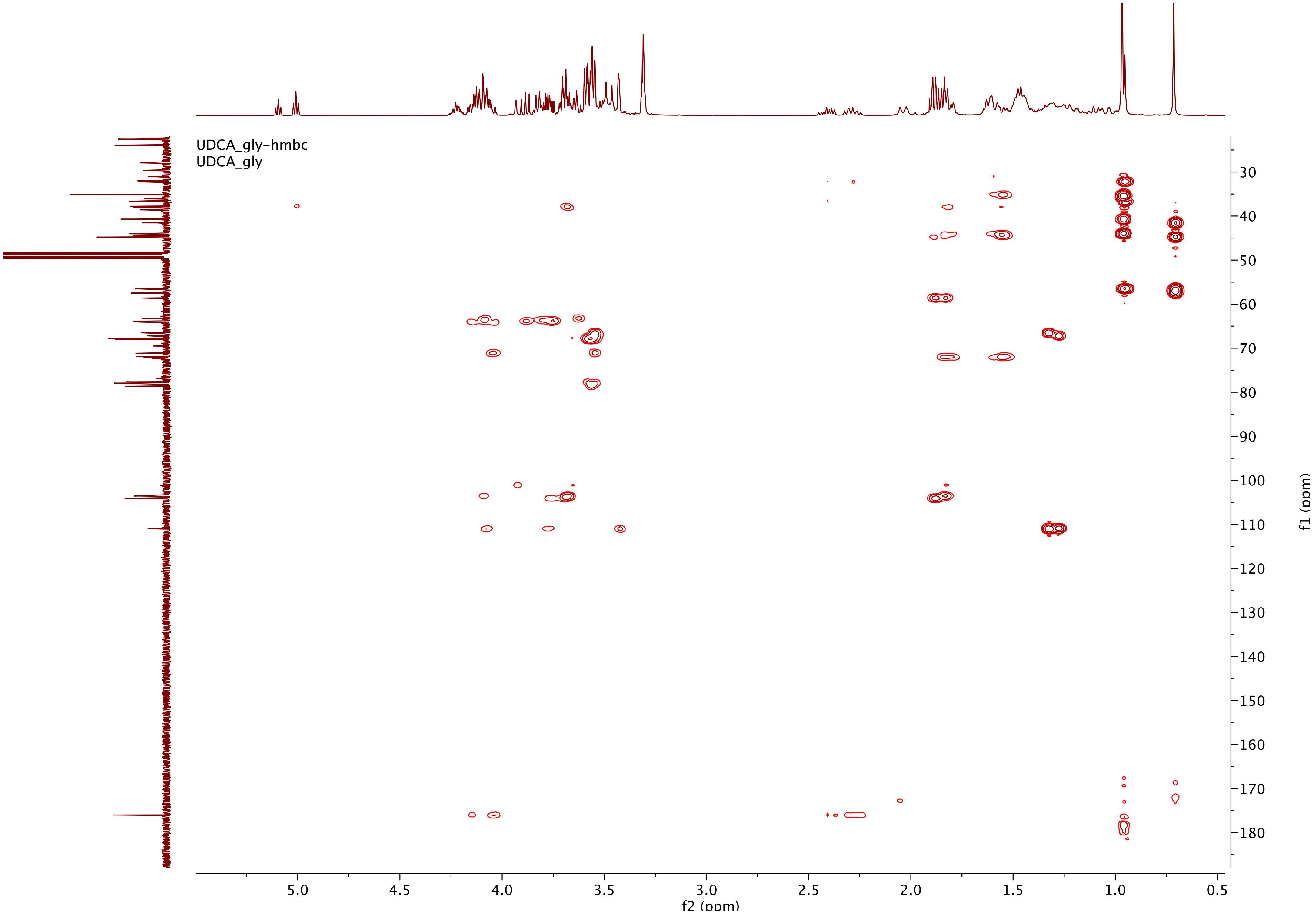
UDCA_gly-hmbc
UDCA_gly
f2 (ppm)
f1 (ppm)

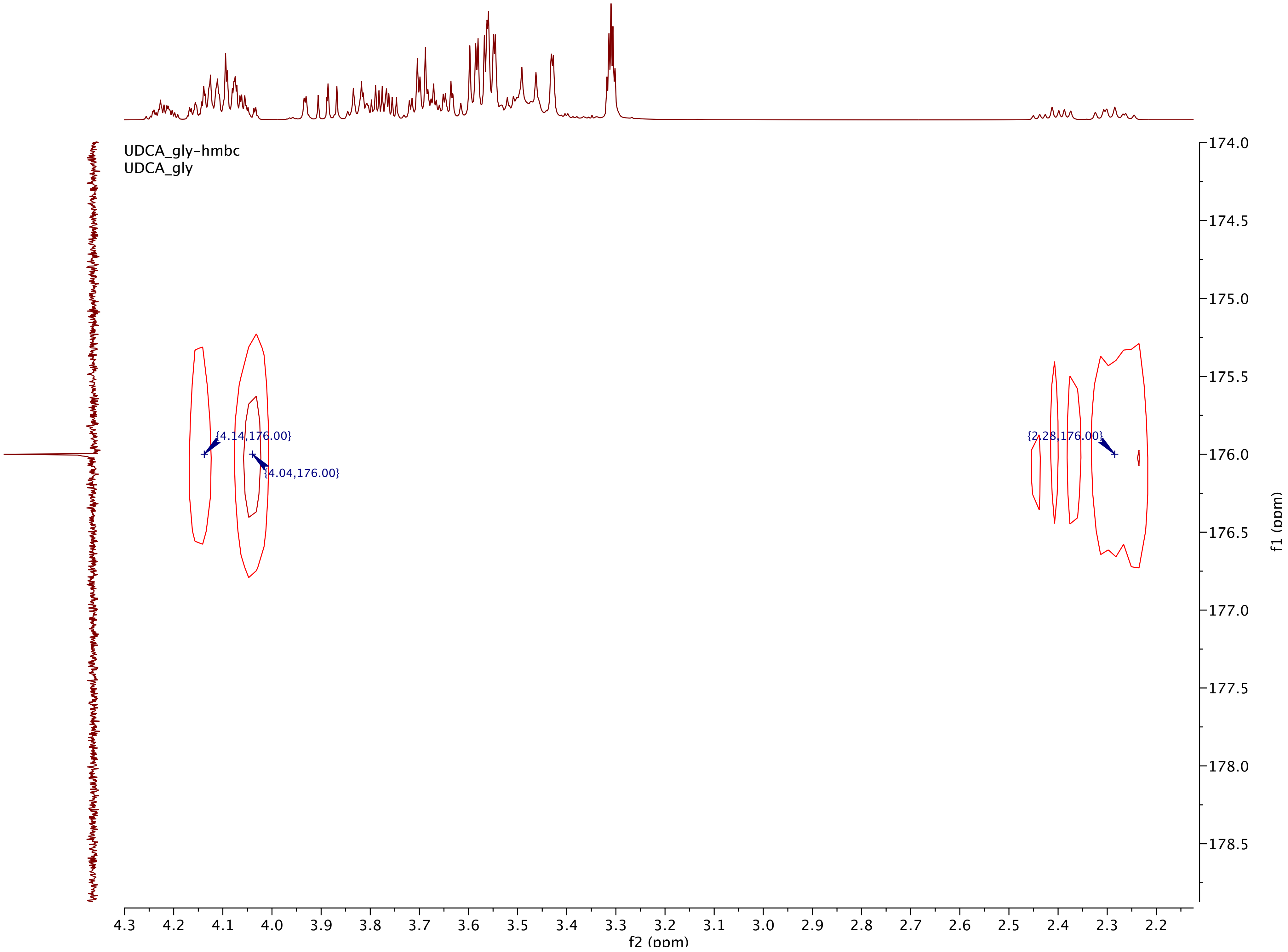

UDCA_gly-hmbc
UDCA_gly
{4.14,176.00}
{4.04,176.00}
{2.28,176.00}
f2 (ppm)
f1 (ppm)

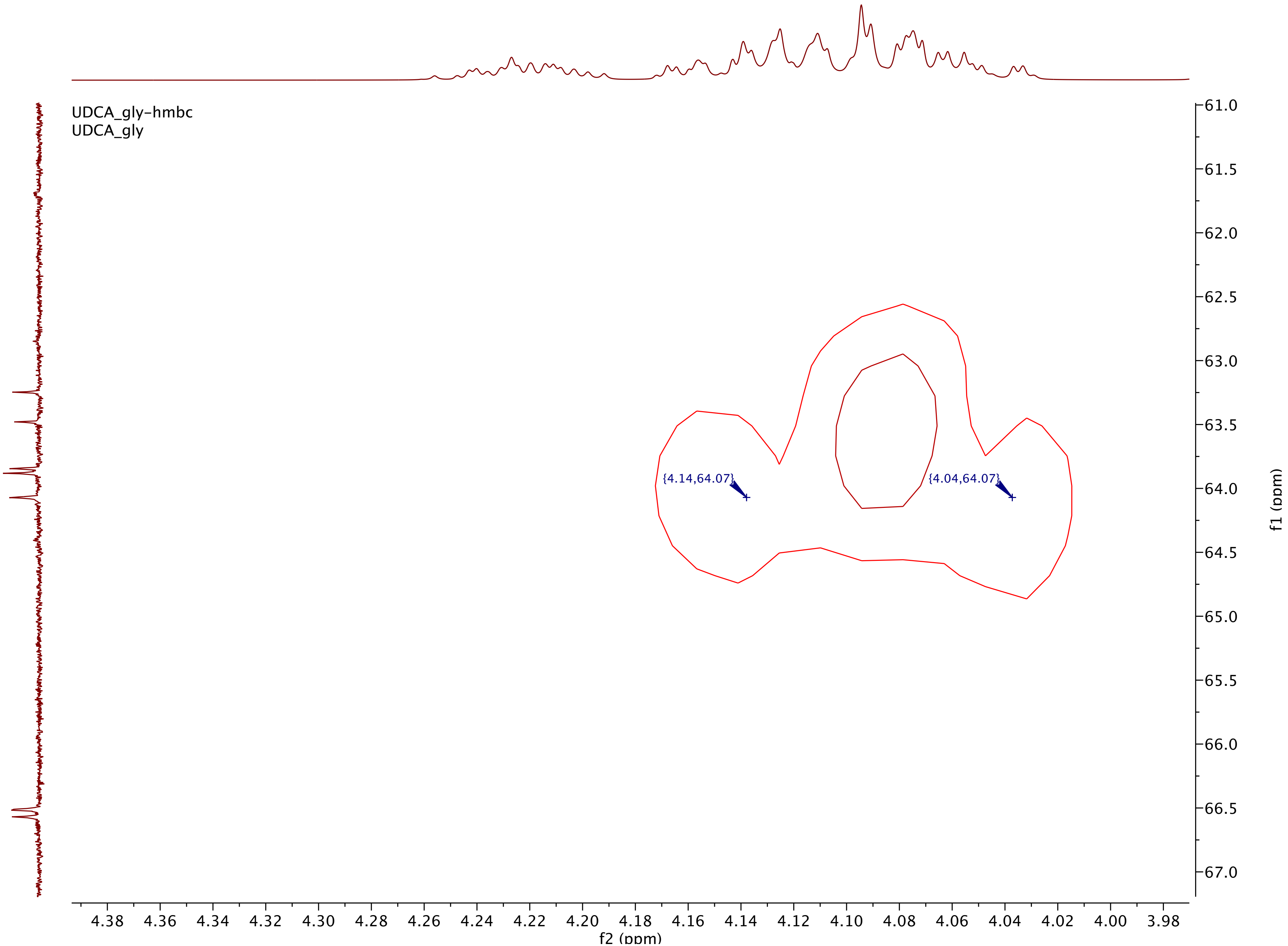
UDCA_gly-hmbc
UDCA_gly
{4.14,64.07}
{4.04,64.07}
f2 (ppm)
f1 (ppm)

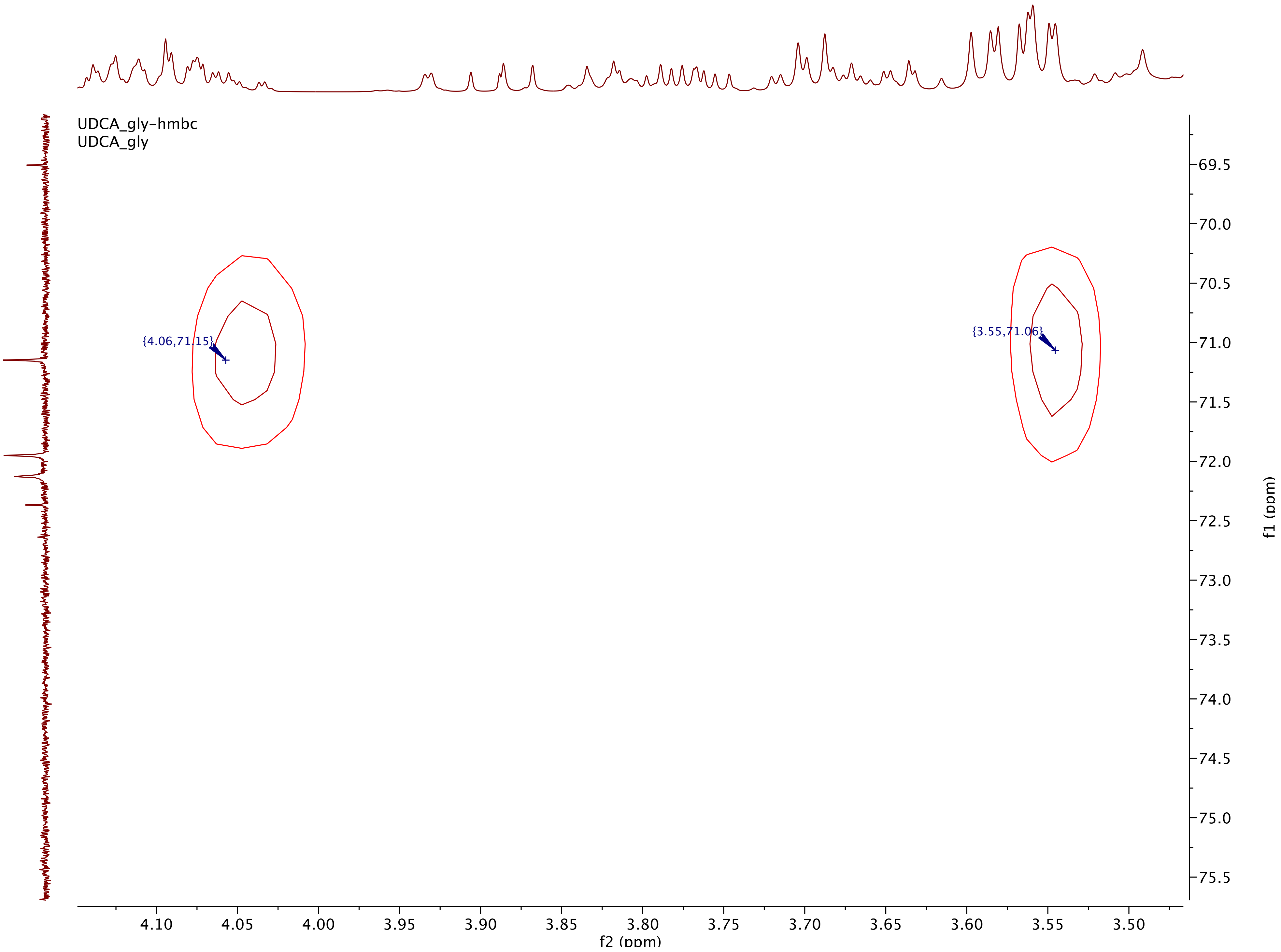

UDCA_gly-hmbc
UDCA_gly
{4.06,71.15}
{3.55,71.06}
69.5
70.0
70.5
71.0
71.5
72.0
72.5
73.0
73.5
74.0
74.5
75.0
75.5
f1 (ppm)
4.10
4.05
4.00
3.95
3.90
3.85
3.80
3.75
3.70
3.65
3.60
3.55
3.50
f2 (ppm)
